# Progression of saproxylic fungal communities in fine woody debris in boreal forests of Oulanka, Finland, assessed by DNA metabarcoding

**DOI:** 10.3897/BDJ.13.e155520

**Published:** 2025-09-25

**Authors:** Maria Shumskaya, Joel Lim, Sarah Apgar, Madhumitha Sadhasivan Gayathri, Adriana Inoa, Dmitry Schigel

**Affiliations:** 1 Kean University, Union, United States of America Kean University Union United States of America; 2 University of Helsinki, Helsinki, Finland University of Helsinki Helsinki Finland

**Keywords:** MycoPins, metabarcoding, GBIF, dead wood, wood decomposition, boreal forest

## Abstract

**Background:**

This publication presents a dataset of saproxylic (dead wood) fungi which addresses the limited understanding of saproxylic fungal diversity, community structure and colonisation dynamics in fine woody debris (FWD). This knowledge gap is largely due to the microscopic or cryptic nature of most fungal species, which often exist primarily as mycelium. To overcome this challenge, the study employs metabarcoding of DNA extracted from pins representing FWD which were experimentally placed for decomposition in northern Finland. The dataset can be used to investigate the composition and progression of fungal communities across different stages of wood decay. It includes communities from distinct biotopes: one forest site protected from reindeer grazing, one exposed to reindeer and a forest area frequented by tourists. The use of standardised decomposition experiments combined with high-throughput eDNA analysis represents a notable methodological approach in characterising saproxylic fungal communities.

**New information:**

The dataset was generated using the novel MycoPins method, published by the authors in 2023, in which sterilised wooden pins were embedded beneath the forest litter and left to decompose for one year, while being periodically inspected. This innovative decomposition experiment was conducted at the Oulanka Research Station in Kuusamo, Northern Finland. As the first study in this region to investigate saproxylic fungi in fine woody debris (FWD) using MycoPins and DNA metabarcoding, it adds new knowledge to the fungal biodiversity data of the region. The resulting dataset of DNA-derived occurrences has been published through the Global Biodiversity Information Facility (GBIF), offering valuable insights into fungal diversity across different stages of decomposition. Agaricomycetes, a class of fungi strongly associated with their hosts via ectomycorrhiza, was selected from the dataset for comparison with fungal datasets from neighbouring regions. Remarkably, the MycoPins method revealed a high proportion of unique Agaricomycetes taxa not captured in existing species checklists from GBIF.org. These findings highlight the study's contribution to advancing biodiversity assessment. The results demonstrate the potential of this approach to enhance our understanding of fungal community dynamics in boreal forest ecosystems.

## Introduction

Forests are crucial ecosystems that support diverse species, classified, based on the dominant tree species present, which in turn determines the forest’s structure, biodiversity and ecological functions (Costanza et al. 2018). Broadleaf forests are composed of deciduous angiosperms, many of which are classified as hardwoods (Yang et al. 2021). In contrast, coniferous forests, populated by gymnosperms, present the majority of softwood trees (Aiba et al. 2013).

Both hardwoods and softwoods contribute to the biodiversity and structural complexity of the ecosystem by providing habitats for various organisms even after the trees have died (Moose et al. 2019, Bradford et al. 2021). The inhabitants are known to differ based on wood characteristics as variable composition of fungal communities in timber can serve as an indicator of the timber's origin (El Sheikha et al. 2013). Dead wood biomass is degraded by saproxylic fungi which convert lignin and cellulose to more simplified forms of carbon, nitrogen and phosphorus, thereby enriching the soil and promoting plant growth (Pasher and King 2009, Větrovský et al. 2014). Variation between fungal communities in gymnosperms and angiosperms has been observed (Krah et al. 2018), with a series of fungal communities emerging at various stages in the decomposition process (Niemelä et al. 1995). The saproxylic inhabitants of deadwood work together to regulate carbon cycles and help buffer environmental change by slowly releasing carbon dioxide into the atmosphere (Triviño et al. 2016).

Human and animal activities also contribute to nutrient and life cycling in the forest ecosystem. In boreal forests of Finland, reindeer are periodically herded, culled and then allowed to re-disperse into the forest. Lichens provide over 80% of the winter diet for these semi-domesticated reindeer (Heggberget et al. 2002) and, accordingly, a decrease in tree and lichen regeneration have been noted in association with this activity (Santalahti et al. 2018). Patterns of fungi consumption related to similar practices in Sweden have been noted by Sami villagers (Inga 2009), but the effects on fungal community diversity and function are only recently being explored as in Santalahti et al. 2018, Ahonen et al. 2021, and Ahonen et al. 2024, and the effects on saproxylic fungi are even less well documented.

Characterisation of deadwood fungal species diversity is complicated by the practical invisibility and, therefore, difficulty of detection of the mycelial stage of the fungal life cycle. Traditional classification has made considerable progress in describing some of the fungal community in dead wood, having elucidated some species-specific biochemical contributions to the decomposition of deadwood (Fukasawa et al. 2017) and described the process of fungal succession in boreal forests (Niemelä et al. 1995). It has also been noted that deadwood fungal diversity has decreased in areas with extensive forest management practices (Ovaskainen et al. 2013). Despite this progress, traditional surveys are limited because they depend on labour-intensive manual efforts, such as examination of fruiting body morphology or identifying species that would grow in culture (Anderson and Cairney 2004). As a result, the investigation of fungi is often restricted to species with readily observable characteristics (Nilsson et al. 2014).

DNA metabarcoding overcomes these difficulties and the method has increased in prominence since the 1990s (Anderson and Cairney 2004). This technique enables assessment of species diversity at a study site based on genetic material that is present in environmental samples. It utilises highly conserved genetic regions as stable reference points adjacent to introns that exhibit considerable interspecific variation and a low degree of intraspecific variation. These regions are used to differentiate amongst species. In fungi, the introns flanking the 5.8S gene, part of the Internal Transcribed Spacer (ITS) region, match this pattern (Schoch et al. 2012). These unique identifiers, once isolated and sequenced, can be compared to sequences of known species held in curated databases, for example, UNITE (Nilsson et al. 2018). This technique is now widely used to rapidly identify large numbers of species present in samples, enabling, amongst others, the characterisation of fungal communities in peatlands (Filippova et al. 2024), in various deadwood source species (Yang et al. 2022), urban vs. semi-natural forests (Korhonen et al. 2022), across time as succession progresses (Lepinay et al. 2021) or even in forensic sciences (Gemmellaro et al. 2023). It should be noted, however, that ITS primers could be biased against certain taxonomic groups, thus DNA metabarcoding using ITS primers may not fully represent all fungal groups in a sample (Bellemain et al. 2010, Mbareche et al. 2021, Kauserud 2023).

Much of what has been characterised in terms of saproxylic species has concentrated on effects noticeable in larger dead fragments or in organisms other than fungi (Ulyshen 2014, Parisi et al. 2018). Recent research has begun a more thorough exploration of the saproxylic fungal communities in fine woody debris (FWD). It has been noted that FWD decay rate increases with temperature (Berbeco et al. 2012), that fungal inhabitants of FWD are host species dependent (Krah et al. 2018b) and that fungal community diversity is positively correlated with volume of FWD (Meyer et al. 2021, Korhonen et al. 2024). Others have begun to illuminate the impact of microclimate on species diversity (Bässler et al. 2010, Krah et al. 2018b, Brabcová et al. 2022); however, on the whole, relatively little has been done in terms of describing the saproxylic fungal inhabitants of FWD, their community structures, their specific roles in the decay process and especially of understanding colonisation dynamics and community development.

**Figure 1.**
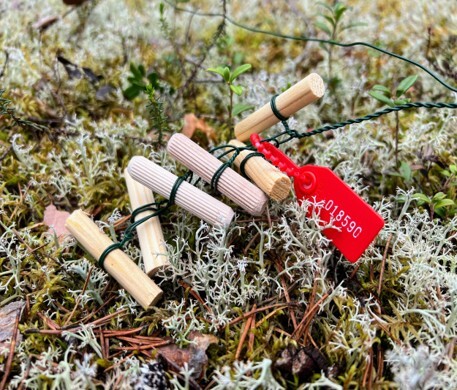
Sterilised and labelled MycoPins sextet prior to depositing under the litter. Each sextet consists of three pairs of identical pins: three tree species in pairs; and a plastic label.

In the presented dataset (Shumskaya et al. 2024), we assessed increasing diversity of saproxylic fungal communities in FWD represented by wooden pins (MycoPins) (Shumskaya et al. 2023), over a period of one year. MycoPins (Fig. 1), made of hardwood and softwood, were placed in sextets of replicated pairs (3 tree species x 2 pairs) in the topsoil of a boreal forest in Finland 2022 in the surroundings of the Oulanka Biological Station and were allowed to decay for one year; every two weeks, a set of pins was removed for analysis. The experiment was set at several different biotopes: conifer forest with and without access by reindeer and a mixed forest to represent differing forest management regimes. The dataset presents fungal communities observed at different stages of decay and in different types of wood (hardwood vs. softwood).

Our experiment presents a controlled field study which was conducted in a forest setting to investigate fungal colonisation of sterilised pins and the progression of fungal communities over time. By placing sterilised pins in the natural environment, we simulate colonisation under controlled conditions, while still allowing natural interactions to occur. This approach enables us to track the establishment and succession of fungal species with precision. We incorporate both positive and negative controls to ensure the reliability of our findings, following standard experimental protocols. Our study provides insights into fungal community dynamics while maintaining the rigour of laboratory experimentation in a natural context. In our dataset, "present" indicates that a species was detected in a particular sampling event using our method, while "absent" means that a species was not detected in a particular sampling event. Notably, if a species is absent in one event, it still may be present in other sampling events of the same transect.

The main goal of this study is to characterise the temporal development and diversity of saproxylic fungal communities in fine woody debris under natural conditions, using a standardised and replicable DNA-based method. By exploring how fungal communities vary across different decay stages, wood types (hardwood vs. softwood) and land-use conditions — including reindeer grazing and tourist access — this research offers novel insights into colonisation dynamics in a largely overlooked substrate. The significance of the study lies in its contribution to our knowledge about fungal biodiversity in boreal ecosystems and in demonstrating how fine-scale molecular monitoring can enhance forest management and conservation efforts.

## General description

### Purpose

**Figure 2.**
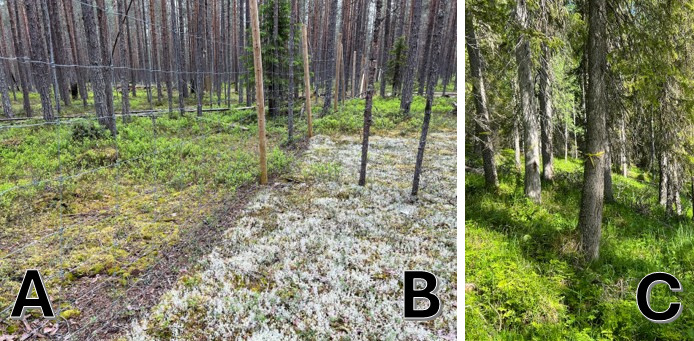
Biotopes representative of transects A, B and C. Abundant *Cladonia* sp. growth is evident in Biotope B, protected from reindeer grazing.

This work elucidates colonisation and community development patterns of saproxylic fungi in undisturbed boreal forests, exploring how environment and forest management practices influence fungal diversity in decaying wood. Leveraging the MycoPins method (Shumskaya et al. 2023), sterilised wooden pins were placed in the topsoil layer and were subjected to natural microbial colonisation with subsequent periodic extraction; fungal colonisation was monitored across three different forest ecosystems near Oulanka Research Station, Kuusamo, northern Finland during 2022–2023. Oulanka Station is a regional unit of the University of Oulu located within Oulanka National Park (66°22’N, 29°19’E, 166.5 m a.s.l.), ca. 25 km south of the Arctic Circle. The station maintains an EcoClimate project which includes a plot guarded from reindeer grazing access for many years. MycoPins were placed at several local biotopes: conifer pine (*Pinus sylvestris* L.) dominated forest with access of reindeer (transect A), conifer pine dominated forest without access of reindeer (transect B) and a mixed, spruce (*Picea abies* (L.) Karst.) dominated forest with birch (*Betula pendula* Roth) and aspen (*Populus tremula* L.) (transect C). Reindeer are a keystone species in boreal forests which defines and shapes the biodiversity of major Nordic ecosystems. *Cladonia* spp. are lichens that are heavily consumed by reindeer and are in abundance in the protected forests and stand excluded from grazing, while suppressed in unprotected forests (Heggenes et al. 2017) (Fig. 2). Hence, reindeer grazing might have an impact on forest microbiome. Our research produced a dataset suitable for testing of several hypotheses: 1) fungal communities develop in newly-available FWD and change subsequently with progression of decay; 2) diversity of saproxylic fungal guilds is different across different biotopes; and 3) fungal colonisation differs in hardwood FWD vs. softwood FWD.

**Figure 3.**
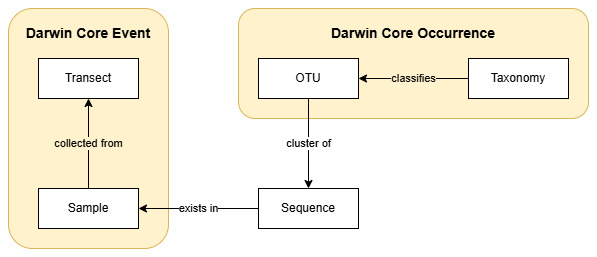
OTU-focused data model used with the MycoPins method. The entities in this study's data model were mapped to the Darwin Core standard (DwC) for publication of the DNA-derived occurrence data through GBIF. The Transect and Sample entities represented Darwin Core Events (parent and child, respectively), while the OTU and Taxonomy entities mapped to the Darwin Core Occurrence terms.

This study adopts a data model (Fig. 3) focused on operational taxonomic units (OTUs), which are defined by clusters of DNA sequences sharing a high degree of similarity and representing specific taxonomic groups. Fungal OTUs were identified using DNA metabarcoding, which analysed fungal DNA from MycoPins undergoing decomposition across three transects over periods ranging from two weeks to one year. A taxonomy was used to classify the cluster of organisms an OTU represents. This study used the SCATA pipeline (https://scata.mykopat.slu.se/) to match each OTU to the UNITE database taxonomy to primarily identify the species; however, for OTUs which were not matched to a UNITE reference by SCATA, a best-effort search from the NCBI database was performed. The top match from the NCBI database search was considered to be the OTUs identity.

**Figure 4.**
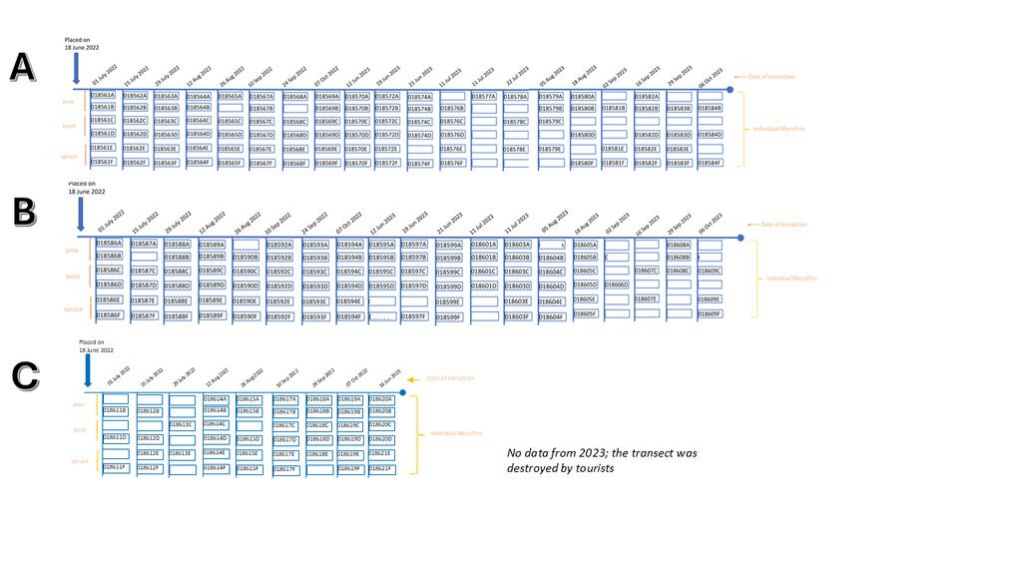
Sampling scheme for MycoPins collected from transects A, B and C. Cells represent individual MycoPins. Empty cells represent MycoPins that were lost during any part of the experimental procedure.

The data model employed in this study was mapped to the Darwin Core Standard (DwC) for GBIF publication as a DNA-derived occurrence dataset. The MycoPins, distributed across various transects, were individually designated as events. Each event was assigned a unique event ID, composed of the transect identifier and the individual sample number. This event ID was further linked to a parent event ID, combining transect identifier with its collection date (Fig. 4). Taxonomic information associated with an OTU within a sample was represented as occurrences. Occurrences were uniquely identified by an occurrence ID, which included the event ID and the GBIF taxon key for the respective fungal species. The representative sequences of OTUs related to each occurrence were captured as DNA data. For example, the event A_018561C pertains to a MycoPin identified as 018561C in transect A. The parent event ID A_2022_Jul_01 refers to a pin collection of transect A that occurred on 1 July 2022. The occurrence ID A_018561C:2613081 represents the *Hormonema macrosporum Voronin* (GBIF taxon key: 2613081) in relation to the event A_018561C.

## Sampling methods

### Study extent

**Figure 5.**
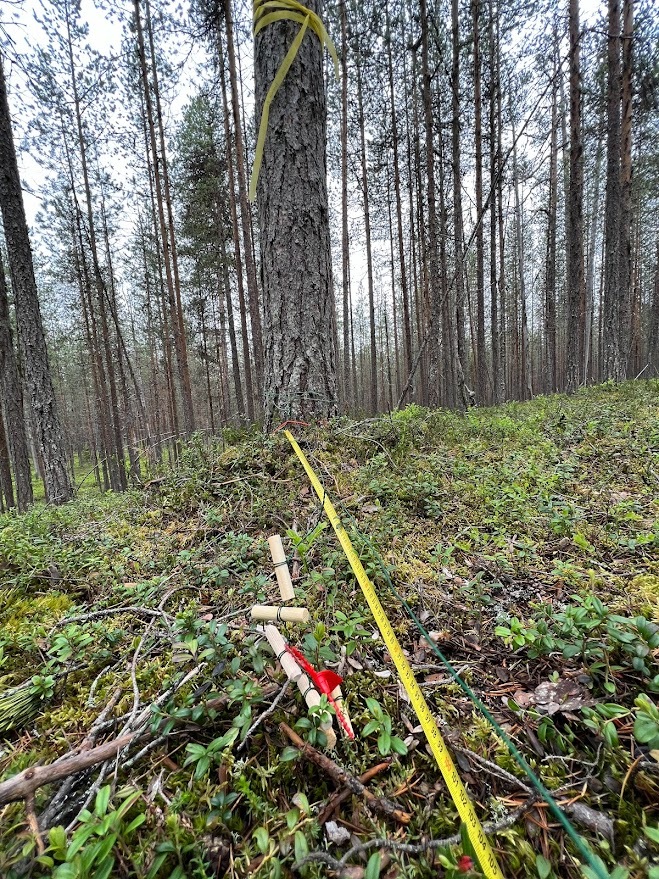
Major wire guide (green) is seen to the right from the yellow measure tape. The beginning of the wire guide is attached to a marked tree for easier detection later. A sextet of MycoPins on a shorter wire is shown branching to the left.

Sampling method followed the MycoPins method described in Shumskaya et al. (2023). The sampling events took place in boreal forests near the Oulanka Biological Station, Finland from 1 July 2022 to 6 October 2023. Sterilised wooden pins made of the xylem of pine, spruce (sourced locally) and birch (purchased from a large hardware store, source unknown), were combined into groups of six (sextets), two per each type of wood and attached with a metal wire to a longer (approximately 20 m) major wire guide which represented a transect, at a distance of 1 m from each other (Fig. 5). Each sextet was labelled with an individual number (generic plastic shipment tags purchased online). Pins within each sextet were labelled later upon collection with letters A to F (A, B - pine, C, D - hardwood, E, F - spruce). Three transects were placed in three different sampling sites with the MycoPin sextets buried under the litter layer. The wire guide was attached at one end to a marked tree and later used to find the sextets.

### Sampling description

**Figure 6.**
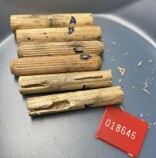
Decomposed MycoPins after collecting, drying and sawdust core extraction.

**Figure 7.**
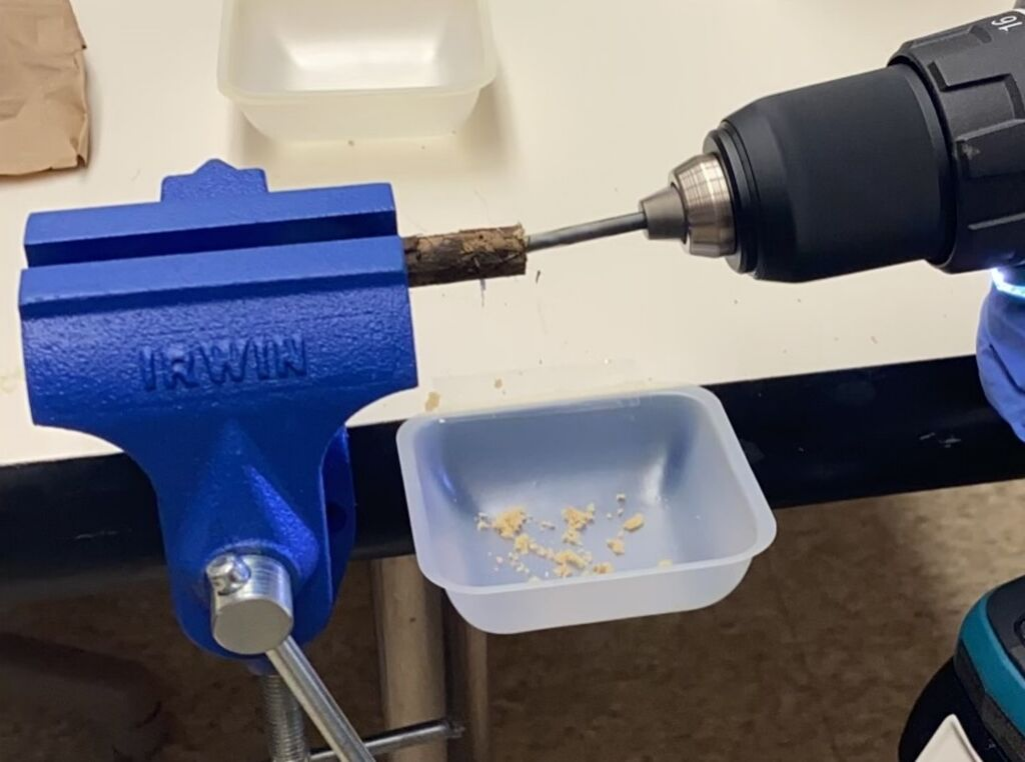
Sawdust extraction from a MycoPin using a fire-sterilised bit.

**Figure 8.**
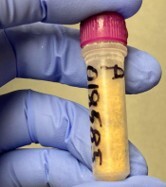
Sawdust extracted from a MycoPin.

Sterilised wooden pins of softwood (pine and spruce) and hardwood (birch) (Fig. 1) were prepared from the xylem of the corresponding trees. Pine and spruce pins were ordered from a local timber shop and birch pins were purchased as furniture pins from a hardware store. Each pin was prepared in a duplicate and then placed on the top soil of three different sampling sites: (A) An area of a pine-dominated boreal forest unprotected from grazing by reindeer; (B) An area of a pine-dominated boreal forest located next to A, but protected from reindeer; a part of the EcoClimate project of the Oulanka Biological Station; and (C) An area of a mixed spruce-dominated forest, accessed by random visitors. One MycoPin sextet from each transect was located using the wire guide and collected every two weeks during summer and autumn seasons between 1 July 2022 and 6 October 2023, with exceptions for weather conditions or instances where individual collectors were unable to conduct sampling. The collected sextets were dried in separate paper bags for 2-3 hours at 45°C and then stored dry at room temperature (Fig. 6). Sawdust was extracted from the core of each pin by drilling (Fig. 7) using fire-sterilised bits and DNA was isolated from the sawdust (Fig. 8).

### Quality control

The MycoPins were sterilised before placement for decay to ensure no fungal species were introduced in the experiment from the outside. To confirm sterility, we isolated DNA and performed PCR for the fungal DNA the same way we did for the decomposed MycoPins. No DNA was isolated from sterilised MycoPins and no fungal ITS PCR product was detected. Hence, we confirm that all species detected in the MycoPins originated from the soil or leaf matter during the process of decay.

Two controls were included in the set up to ensure quality of DNA analysis. A positive control of SynMock artificial fungal community (Palmer et al. 2018) made of 0.1 ng of a mix of 12 plasmids with ITS inserts served as an internal positive control for metabarcoding procedures which include PCR, next generation sequencing and bioinformatics procedures. This control ensured that we could reliably identify a fungal species at a low concentration of DNA. Negative control (water) was also used in the same PCR, next generation sequencing and bioinformatics procedures to exclude any contamination from laboratory settings or materials.

For bioinformatic analysis, we used paired FASTQ files from which we trimmed sequences with low quality. Curated UNITE Fungi v.9.0 (2023-07-18) (Abarenkov et al. 2023a) database was used for species identification as a first choice. For sequences that SCATA was unable to match with a reference in the UNITE Fungi database, additional identification was performed using BLASTn searches against NCBI GenBank (https://www.ncbi.nlm.nih.gov/genbank/). Taxonomic assignments were cross-referenced with the GBIF Backbone Taxonomy to resolve synonyms and ensure consistency. Only species of the Fungi kingdom were included in the species list.

### Step description

**DNA isolation.** The core of each pin from each sextet was drilled using a 2 mm fire-sterilised drill bit. The resultant sawdust was collected in a sterile centrifuge tube (Fig. 8). The sawdust was then used to isolate genomic DNA using PowerSoil DNA Isolation Kit from Qiagen (USA) according to the manufacturer's instructions. Homogenisation of the sawdust with the DNA extraction buffer from the kit was performed using BeadBug homogenisers (BenchMark Scientific). DNA concentration was measured using NanoDrop (ThermoFisher). Genomic DNA was stored at −80°C.

**PCR.** A set of 40 tagged primers for the ITS2 fungal region (Clemmensen et al. 2016) was used for PCR of each DNA sample. Tagged primers were designed as follows: while the forward and reverse primers were always the same (fITS7 GTGARTCATCGAATCTTTG and ITS4 TCCTCCGCTTATTGATATGC), the primers were modified to include a unique 10-nucleotide tag as described in Clemmensen et al. (2016) to create 40 unique primer pairs. A tagged fITS7-ITS4 pair was used to perform PCR for the DNA extracted from each MycoPin. The amplification was verified via agarose gel electrophoresis (Suppl. material 1). The amplified DNA was purified and stored at −20°C. E.Z.N.A® Cycle Pure Kit (Omega Bio-tek) was used for the purification of the amplicons. A positive control was used to verify the PCR and subsequent next generation sequencing in a form of mock fungal community (SynMock) made of 0.1 ng of a mix of 12 plasmids (Palmer et al. 2018). A negative control (water) was used in the same way to exclude false-positive results.

**Next-Generation Sequencing.** Equal amounts of tagged amplicons (100 ng each) were then mixed in groups of 40 according to the unique, non-repeated tags, into a multiplex, which was used for sequencing. The multiplexes were sequenced using AmpliconEZ service at a commercial facilty Genewiz (Azenta Life Sciences, New Jersey, USA; at Azenta/GeneWiz, DNA library preparation was performed using NEBNext Ultra DNA Library Prep kit (Illumina, USA), DNA libraries were validated on the Agilent Tape Station (Agilent Technologies, USA) and quantified using Qubit 2.0 fluorimeter (Thermo Fisher, USA), the pooled DNA libraries being loaded on the Illumina MiSeq instrument and sequenced using a 2× 250 paired-end configuration). The sequencing results were returned as two FASTQ files for each sequenced multiplex. Raw reads (FastQ files) were uploaded to NCBI Sequence Read Archive (BioProject accession number PRJNA1294490).

**Bioinformatics.** Two paired FASTQ files for each multiplex were analysed using the following procedure:

1. The FASTQ files were uploaded to the SCATA pipeline (https://scata.mykopat.slu.se).
2. The SCATA pipeline was used to exclude low quality sequences, cluster similar sequences, sequences without tags and identify species using UNITE Fungi v.9.0 (18-07-2023) (Abarenkov et al. 2023a) fungi database.
  - Sequence quality was parameterised to include only the following: (1) a 90% primer match on tag identification; (2) a minimum sequence length of 200; (3) a minimum base quality of 10; and (4) a minimum mean base quality of 20. Pipeline was configured to overlap and merge the FASTQ files. Kmer size for overlap search was set to 7. The minimum number of adjacent kmers to form high-scoring segment pairs during overlap search was set to 5. The minimum number of shared kmers to merge a read pair was set to 10;
  - SCATA uses the USEARCH algorithm for clustering. Clustering distance was set to 0.015. The minimum proportion of the longest sequence in a sequence pair to consider for clustering was set to 0.85. The penalty for mismatch was set to 1. No penalty was set on an introduction of an open gap. However, a penalty of 1 is incurred for each succeeding gap. No weights were used for end gaps. Homopolymers longer than 3 before clustering were collapsed. No downsampling and no removal of low frequency genotypes were performed during clustering. Up to 3 representative sequences were reported for each cluster.
3. Singletons, double clusters and clusters present in positive and negative controls were excluded.
4. On occasion, SCATA was unable to find a match in the UNITE database. In this case, a BLASTn search was performed for such OTU against the NCBI database. The best match was determined, based on the lowest e-value and highest percentage identity, excluding any results with a BLAST score below 200. If multiple top matches were found, the first listed was selected. All selected matches had e-values below 1E-50 and percent identities above 85%. The read counts of OTUs identified as the same species via NCBI were then combined to reflect their total representation in the dataset.
5. An OTU was assigned a normalised taxonomic name, based on its source taxonomic name (UNITE or NCBI). The normalisation rules were as follows:

- Remove all text from “ sp.” until the end. For example, the normalised name for “*Peniophora* sp.” will be “*Peniophora*";
- Remove “uncultured“, if the source taxonomic name starts with “uncultured”. For example, the normalised name for “uncultured *Trechispora*” will be “*Trechispora*”;
- A normalised name of “fungus” is assigned to the source taxonomic name that starts with the word “fungal”. These are typically taxonomic names coming from NCBI.

1. Each OTU was aligned with the GBIF Backbone taxonomy using statistical software R and rgbif package v. 3.7.9 (Chamberlain et al. 2012) . Using the normalised name (#5 above), we used the GBIF backbone API to determine the GBIF accepted name. If the GBIF accepted name were not available, we used the GBIF original name. We opted to use the GBIF scientific name as it provides uniformity and is determined by the normalised name of the source taxonomy. Additionally, in this published dataset, we maintained the UNITE species hypothesis or NCBI taxonomy ID as taxonConceptID of an occurrence. This way, the species identity associated with the occurrence is less ambiguous and more specific.
2. Non-fungal species were rejected. Fungal species not identified at the phylum level, at the minimum, were also discarded. Fungal traits were assigned according to the FungalTraits database (from Supplementary Data of Põlme et al. (2021)). Historical weather data for each transect were gathered from Weatherstack (www.weatherstack.com).

## Geographic coverage

### Description

Oulanka Research Station (www.oulu.fi/en/research/research-infrastructures/oulanka-research-station and https://eu-interact.org/field-sites/oulanka-research-station) is located in Kuusamo, northern Finland, 25 km south of the Arctic Circle, Sub-Arctic (Boreal zone), no permafrost.

### Coordinates

 and 66°22'12.0" Latitude; and 29°18'34.7" Longitude.

## Taxonomic coverage

### Description

Saproxylic fungi belonging to the Ascomycetes and Basidiomycetes were identified from DNA extracted from sawdust obtained from wooden pins made of pine (*Pinus sylvestris* L), spruce (*Picea abies* (L.) Karst.) and birch (*Betula pendula* Roth), using the MycoPins method (Shumskaya et al. 2023). Analysis of the dataset revealed a total of 3,990 operational taxonomic units (OTUs), which were classified into 215 species, 89 genera, 22 families, 18 orders, 8 classes, 2 phyla and one kingdom, based on a 98.5% sequence similarity threshold. Of these, approximately 52.28% of OTUs were identified at the species level and 25.41% at the genus level (Table 1).

It is important to note that species-level identifications inherently include genus-level identification and the reported percentage for genus-level identifications refers specifically to those OTUs identified at the genus level only, not including those resolved to species. In terms of relative read abundance, OTUs identified at the species level accounted for approximately 52.28% of the total reads, while those identified only at the genus level contributed 25.41%, indicating that most of the sequencing reads were assigned to OTUs that could be identified at the species level.

Datasets spanning multiple regional scales, as presented in Table 2, were obtained from GBIF.org and verified for accuracy to ensure consistency in species names across regional lists. We used class Agaricomycetes for this analysis since it was well represented in our dataset (MycoPins Oulanka National Park). Good representation is likely related to the fact that Agaricomycetes are closely ectomycorrhizally associated with their hosts (Sato 2023). Checklists of preserved Agaricomycetes specimens were compiled for comparison, filtered by record type (“preserved specimen”), occurrence status (“present”) and collection years (2022–2023) across three regional scales: Oulanka National Park, Finland and the Nordic Region. Our analysis found that approximately half of the species in our dataset were also recorded at the largest scale, the Nordic Region. In contrast, only six species overlapped between our dataset and the GBIF records for Oulanka National Park. The discrepancy, with 41 species found exclusively in our dataset, is likely attributable to the study's specific focus on fine woody debris and the eDNA-based metabarcoding methodology used.

### Taxa included

**Table:** 

Rank	Scientific Name	
kingdom	Fungi	

## Temporal coverage

**Data range:** 2022-6-18 – 2023-10-06.

## Usage licence

### Usage licence

Other

### IP rights notes

This work is licensed under Creative Commons Attribution (CC-BY) 4.0 Licence.

## Data resources

### Data package title

Saproxylic fungi of fine woody debris, studied by metabarcoding-based MycoPins method in Oulanka, Finland, 2022-2023

### Resource link

https://doi.org/10.15468/yfemwn

### Alternative identifiers

www.gbif.org/dataset/63283fef-d82f-40ba-9346-c4810e9690dc

### Number of data sets

1

### Data set 1.

#### Data set name

DNA-derived Occurrence dataset

#### Data format

Darwin Core

#### Character set

UTF-8

#### Download URL

https://www.gbif.org/occurrence/download?dataset_key=63283fef-d82f-40ba-9346-c4810e9690dc

#### Description

**Figure 9.**
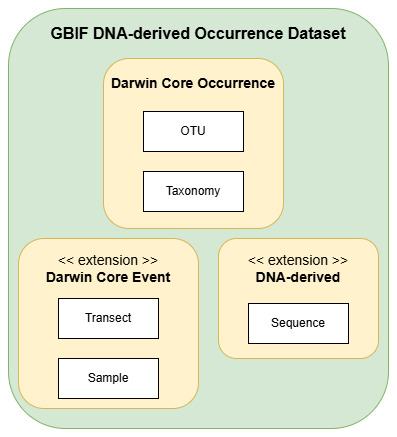
High-level view of the GBIF DNA-derived occurrence dataset generated from the MycoPins method data model, mapped as Darwin Core standard and extension.

The dataset represents DNA-based occurrences in a form of an occurrence dataset in GBIF, prepared following the guide to publishing DNA-derived data through biodiversity data platforms (Abarenkov et al. 2023b). The dataset is derived from the data model for the MycoPins method mapped to the Darwin Core standard (Fig. 9).

**Figure 10.**
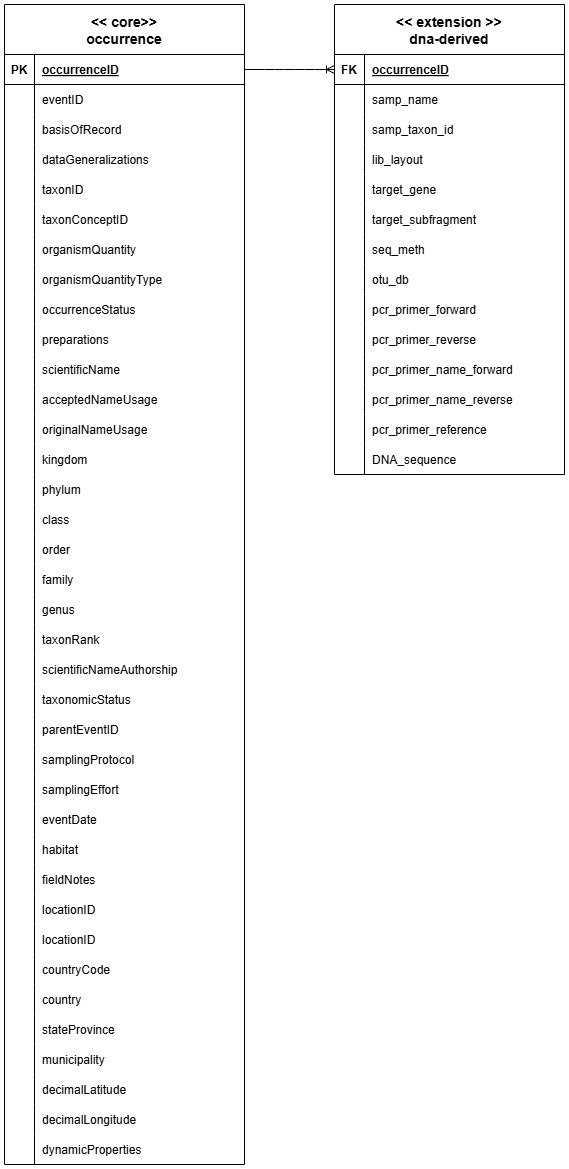
Structure of the DNA-derived occurrence dataset from this study.

The dataset includes a DNA-derived extension table and comprises two primary components (Shumskaya et al. 2024). The first, the occurrence table (Occurrence core) documents taxonomic occurrences — presented in two forms: GBIF interpreted and verbatim — as well as information on abundance and presence/absence. It also contains associated event metadata. This structure enables the dataset to be displayed in an event-based format, while incorporating the DNA-derived data extension of the Darwin Core standard. The second table, the DNA-derived data (DNA-derived extension), contains DNA sequences linked to each occurrence (Fig. 10).

**Data set 1.** 

Column label	Column description
occurrenceID (dwc:Occurrence)	A globally unique identifier for an occurrence in an event.
eventID (dwc:Event)	An identifier of an event associated with the occurrence.
basisOfRecord (dwc:Occurrence)	The specific nature of the data record ("MaterialSample").
dataGeneralizations (dwc:Occurrence)	Information on the method of species identification of an occurrence.
taxonID (dwc:Occurrence)	An identifier for the set of taxon information.
taxonConceptID (dwc:Occurrence)	An identifier for the taxonomic concept to which the record refers. Two taxonomy sources are supported, UNITE and NCBI. For occurrences identified through a UNITE database match, the species hypothesis id is provided. Otherwise, the occurrences are matched with the NCBI database and NCBI taxonomy id is provided.
organismQuantity (dwc:Occurrence)	A number or enumeration value for the quantity of organisms.
organismQuantityType (dwc:Occurrence)	Type of organism quantity ("DNA sequence reads").
occurrenceStatus (dwc:Occurrence)	The presence or absence of a taxon at a location.
preparations (dwc:Occurrence)	Preparations and preservation methods ("DNA extract from a MycoPin").
scientificName (dwc:Occurrence)	The full scientific name of a taxon.
acceptedNameUsage (dwc:Occurrence)	The full name of the currently valid (zoological) or accepted (botanical) taxon.
originalNameUsage (dwc:Occurrence)	The taxon name as it originally appeared when first established under the rules of the associated nomenclature.
kingdom (dwc:Occurrence)	The full scientific name of the kingdom in which the taxon is classified.
phylum (dwc:Occurrence)	The full scientific name of the phylum or division in which the taxon is classified.
class (dwc:Occurrence)	The full scientific name of the class in which the taxon is classified.
order (dwc:Occurrence)	The full scientific name of the order in which the taxon is classified.
family (dwc:Occurrence)	The full scientific name of the family in which the taxon is classified.
genus (dwc:Occurrence)	The full scientific name of the genus in which the taxon is classified.
taxonRank (dwc:Occurrence)	The taxonomic rank of the most specific name in the scientific name.
scientificNameAuthorship (dwc:Occurrence)	The authorship information for the scientific name.
taxonomicStatus (dwc:Occurrence)	The status of the use of the scientific name as a label for a taxon.
parentEventID (dwc:Event)	An identifier for the broader event that groups this and potentially other events.
samplingProtocol (dwc:Event)	Reference to the methods or protocols used during an event. ("https://doi.org/10.3897/mycokeys.96.101033")
samplingEffort (dwc:Event)	The amount of effort expended during an event.
eventDate (dwc:Event)	The date-time or interval during which an event occurred.
habitat (dwc:Event)	Description of the habitat in which the event occurred.
fieldNotes (dwc:Event)	The text of notes taken in the field about the event.
locationID (dwc:Event)	Identifier for location in which the event occurred ("https://www.geonames.org/12226273")
countryCode (dwc:Event)	Standard code for the country in which the event occurred ("FI").
country (dwc:Event)	Country in which the event occurred ("Finland").
stateProvince (dwc:Event)	State, province or region in which the event occurred ("North Ostrobothnia").
municipality (dwc:Event)	City or municipality in which the event occurred ("Kuusamo").
decimalLatitude (dwc:Event)	The geographic latitude of the geographic centre in which the event occurred.
decimalLongitude (dwc:Event)	The geographic longitude of the geographic centre in which the event occurred.
dynamicProperties (dwc:Event)	Additional information on the occurrence. This includes the wood type, wood texture, weather data, species identification method details and FungalTraits information. The data is in JSON format.
samp_name (DNA-derived)	MycoPin sample number.
samp_taxon_id (DNA-derived)	UNITE or NCBI taxon id of the sample.
lib_layout (DNA-derived)	Reads configuration ("paired").
target_gene (DNA-derived)	Targeted gene ("ITS").
target_subfragment (DNA-derived)	Name of subfragment of a gene ("ITS2").
seq_meth (DNA-derived)	Sequencing method used ("Illumina MiSeq").
otu_db (DNA-derived)	Reference database ("Clustering based on the UNITE Fungi v.9.0 (18-07-2023) (https://doi.org/10.15156/BIO/2938068) using USearch with 90% identity parameter (SCATA)").
pcr_primer_forward (DNA-derived)	Tagged forward PCR primer.
pcr_primer_reverse (DNA-derived)	Tagged reverse PCR primer.
pcr_primer_name_forward (DNA-derived)	Name of the forward PCR primer without a tag ("fITS7").
pcr_primer_name_reverse (DNA-derived)	Name of the reverse PCR primer without a tag ("ITS4").
pcr_primer_reference (DNA-derived)	Reference for the PCR primers ("https://doi.org/10.1007/978-1-4939-3369-3_4").
DNA_sequence (DNA-derived)	The DNA sequence (OTU). Three sequences per OTU are provided because this is the outcome provided by SCATA pipeline (https://scata.mykopat.slu.se/).

## Additional information

### Conclusion

This paper presents DNA metabarcoding data on saproxylic fungal communities inhabiting fine woody debris (FWD) in boreal forests of northern Finland. The dataset, based on a controlled decomposition experiment using sterilised hardwood and softwood pins (MycoPins), was collected over one year and includes samples from multiple forest biotopes with varying exposure to reindeer grazing and human activity. The data are published through the open-access repository GBIF.org. The experimental setup, sampling design and bioinformatic pipelines used for species identification are described in detail. This study uses a novel and replicable approach for monitoring fungal colonisation dynamics in FWD, contributing insights into fungal biodiversity and the impact of land-use practices on cryptic decomposer communities. Expansion of such datasets will improve our understanding of fungal succession patterns, functional roles of saproxylic fungi and ecosystem responses to climate or forest management changes.

## Supplementary Material

28D22F3E-C129-5560-B974-4C1021F81E0E 10.3897/BDJ.13.e155520.suppl1 Supplementary material 1 Gel electrophoresis of DNA samples extracted from all MycoPins included in the published dataset Data type images of gel electrophoresis Brief description The file contains original images of gel electrophoresis used to verify the PCR amplification of the ITS region from DNA extracted from each MycoPin included in the dataset. https://doi.org/10.15468/yfemwn File: oo_1353952.pdf https://binary.pensoft.net/file/1353952 Shumskaya M, Lim J, Apgar S, Gayathri M.S, Inoa A, Schigel D.

## Figures and Tables

**Table 1.** Taxonomic resolution of saproxylic fungi, identified through eDNA sequencing at Oulanka Research Station, Finland.

	Kingdom	Phyla	Class	Order	Family	Genus	Species
Total number of taxa	1	2	8	18	22	89	215
Number of OTUs identified to this level	55	155	228	247	205	1014	2086
Percentage of OTUs identified to this level	1.38%	3.88%	5.71%	6.19%	5.14%	25.41%	52.28%

**Table 2.** Species counts for Agaricomycetes datasets published at GBIF.org. MycoPins Oulanka National Park: from the dataset of this study (GBIF.Org User 2025a). GBIF Oulanka National Park: the dataset of preserved specimens for Oulanka National Park (GBIF.Org User 2025b). GBIF Finland: the dataset of preserved specimens for Finland (GBIF.Org User 2025c). GBIF Nordic: the dataset of preserved specimens of the Nordic Region (GBIF.Org User 2025d). Total species counts are presented on the diagonal. Unique or shared species numbers are presented above the diagonal.

Dataset name (shared/unique species)	MycoPins Oulanka National Park	GBIF Oulanka National Park	GBIF Finland	GBIF Nordic
MycoPins Oulanka National Park	**47 species**	MycoPins Oulanka National Park unique: 41 GBIF Oulanka National Park unique: 94 Shared: 6	MycoPins Oulanka National Park unique: 21 GBIF Finland unique: 1696 Shared: 26	MycoPins Oulanka National Park unique: 19 GBIF Nordic unique: 2624 Shared: 28
GBIF Oulanka National Park		**100 species**	GBIF Oulanka National Park unique: 0 GBIF Finland unique: 1622 Shared: 100	GBIF Oulanka National Park unique: 0 GBIF Nordic unique: 2552 Shared: 100
GBIF Finland			**1722 species**	GBIF Finland unique: 0 GBIF Nordic unique: 930 Shared: 1722
GBIF Nordic				**2652 species**
