# Supplementary material for: Progression of saproxylic fungal communities in fine woody debris in boreal forests of Oulanka, Finland, assessed by DNA metabarcoding
Source: Biodivers Data J. 2025 Sep 25;13:e155520. doi: 10.3897/BDJ.13.e155520 (PMC12491977; doi:10.3897/BDJ.13.e155520)
Supplement: Supplementary material 1 — Gel electrophoresis of DNA samples extracted from all MycoPins included in the published dataset [file bdj-13-e155520-s001.pdf]

Supplementary data

Shumskaya M, Lim J, Apgar S, Gayathri M.S, Inoa A, Schigel D. (2025) Progression of saproxylic fungal communities in fine woody debris in boreal forests of Oulanka, Finland, assessed by DNA metabarcoding. Biodiversity Data Journal

Gel electrophoreis of DNA samples extracted from all MycoPins from the published the dataset (https://doi.org/10.15468/yfemwn). Numbers correspond to the individual numbers of each MycoPin (Fig. 4).

1st well is DNA marker: 1 kb DNA Ladder (Promega, USA), size in bp. Note that these are the original gels; unlabeled wells contain materials unrelated to this publication.

Transect A

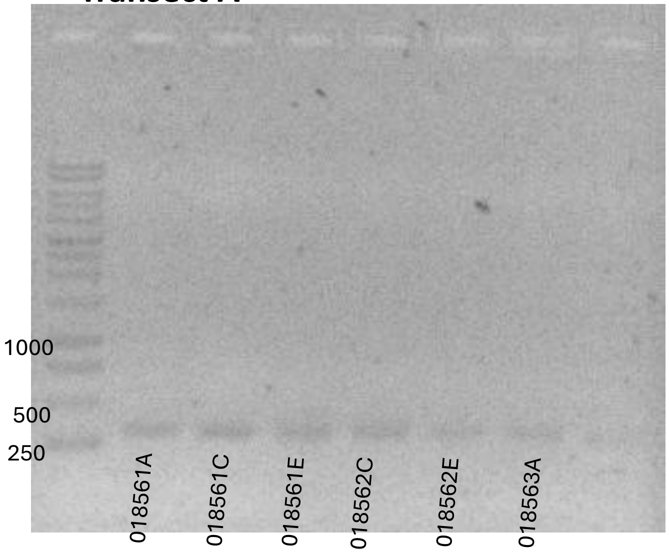

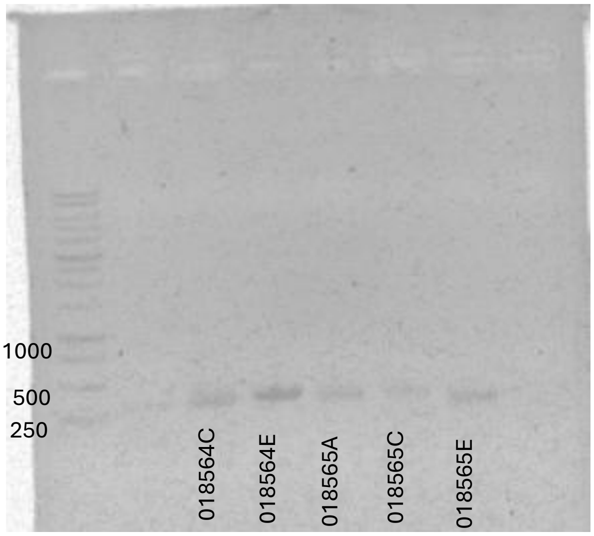

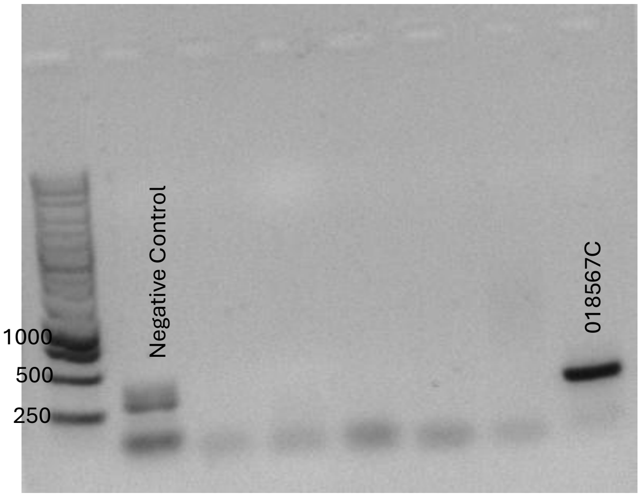

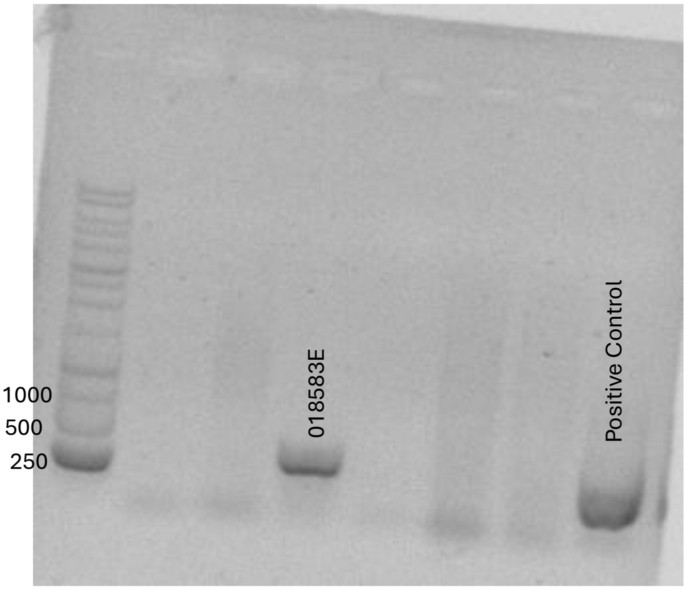

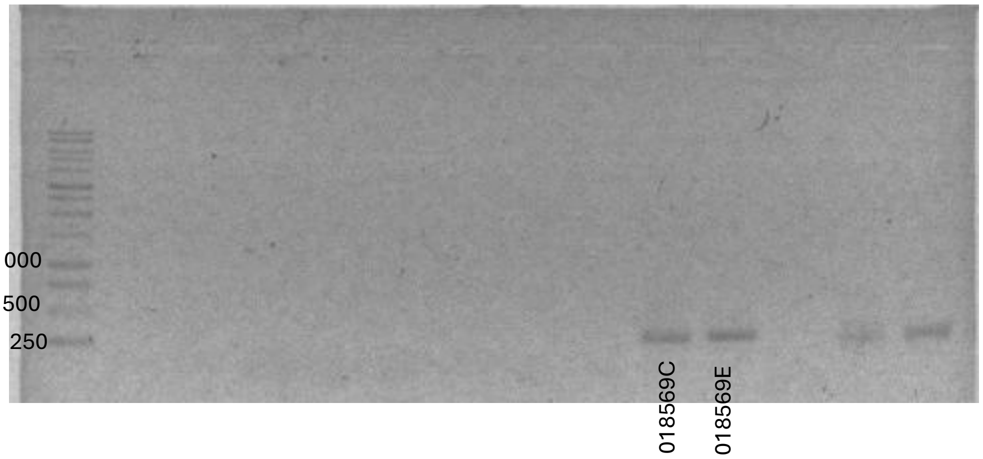
1000
500
250
018569C
018569E

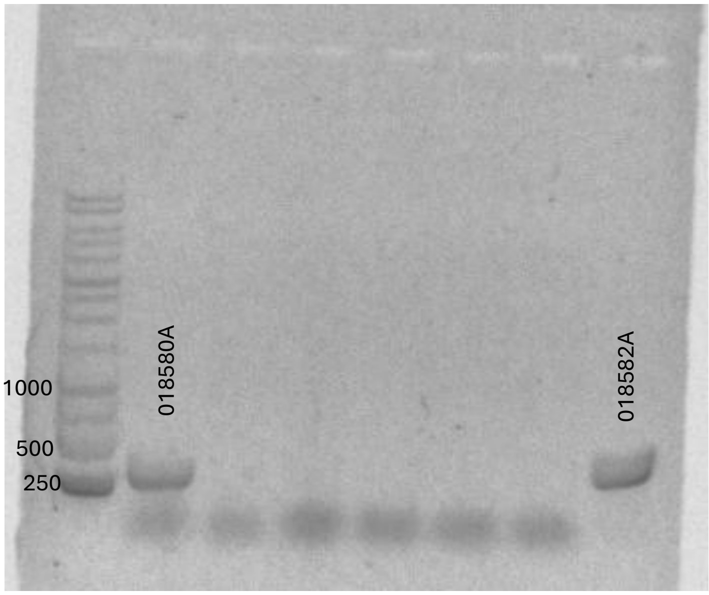
1000
500
250
018580A
018582A

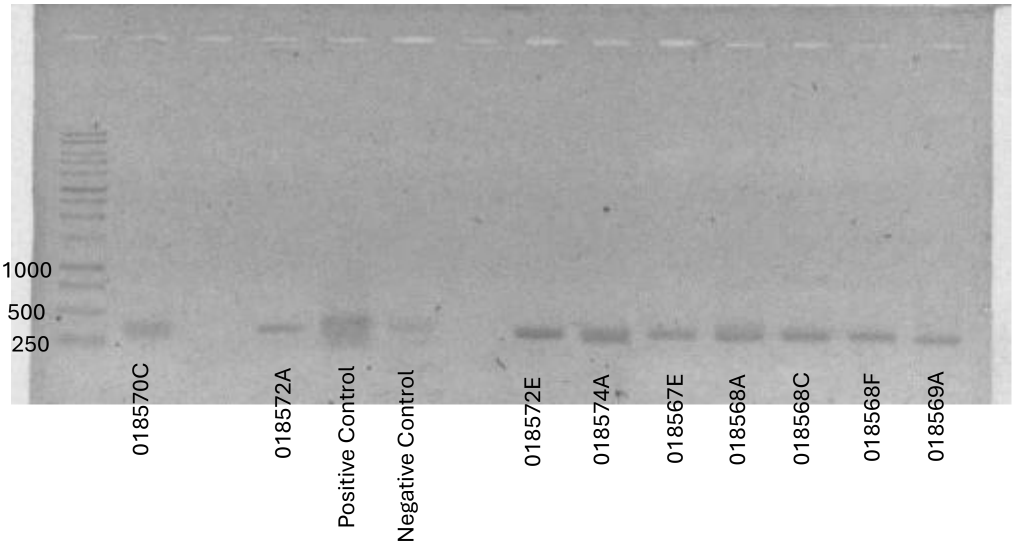
1000
500
250
018570C
018572A
Positive Control
Negative Control
018572E
018574A
018567E
018568A
018568C
018568F
018569A

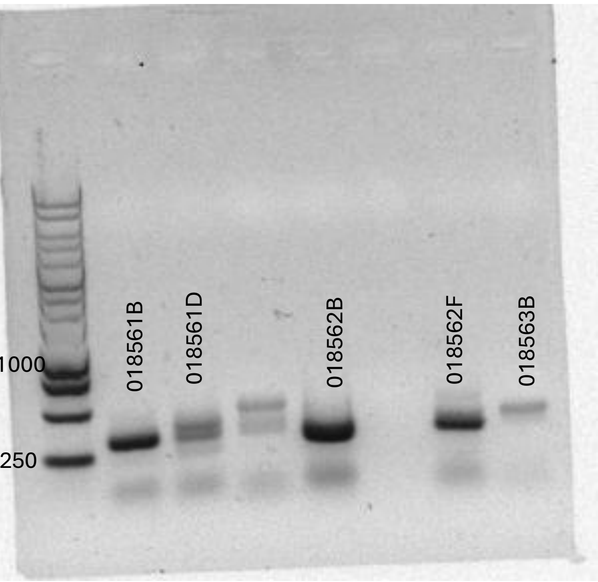
1000
250
018561B
018561D
018562B
018562F
018563B

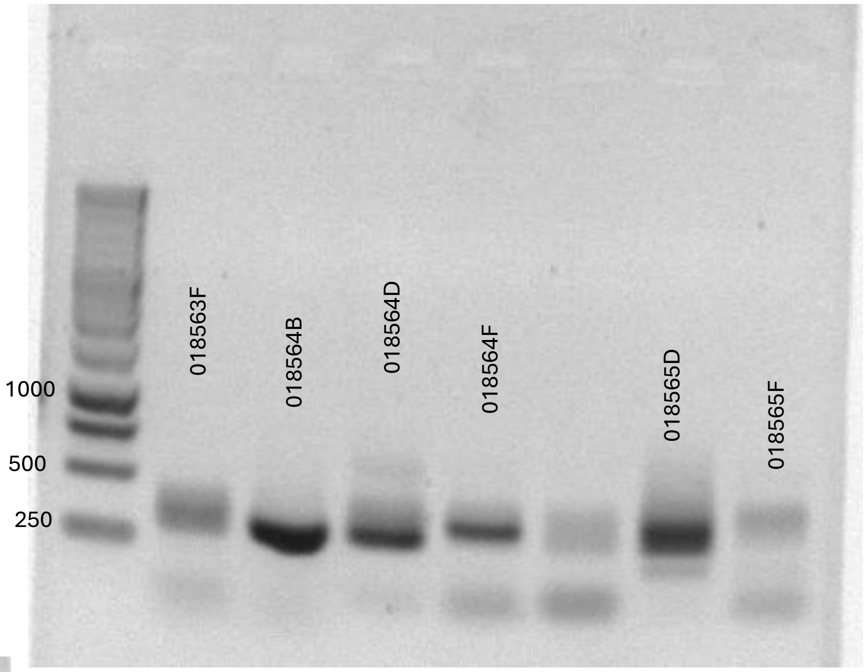
1000
500
250
018563F
018564B
018564D
018564F
018565D
018565F

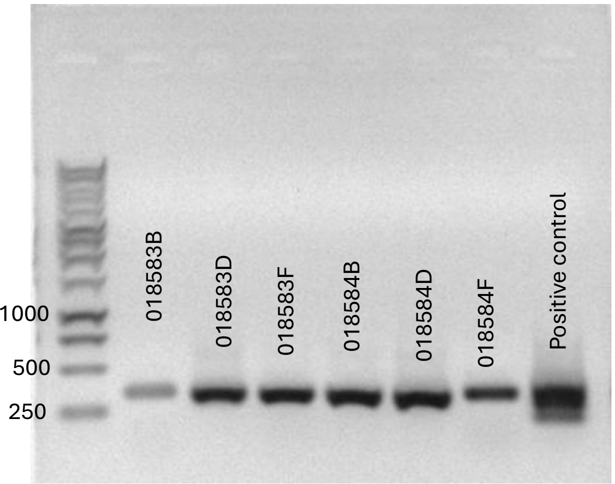
1000
500
250
018583B
018583D
018583F
018584B
018584D
018584F
Positive control

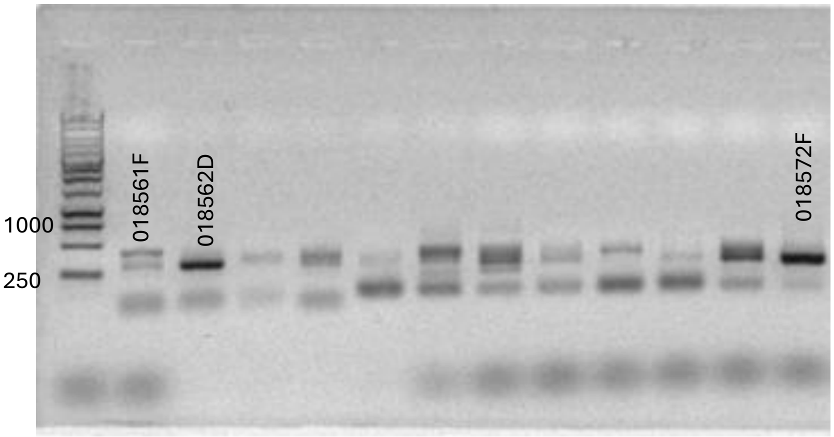
1000
250
018561F
018562D
018572F

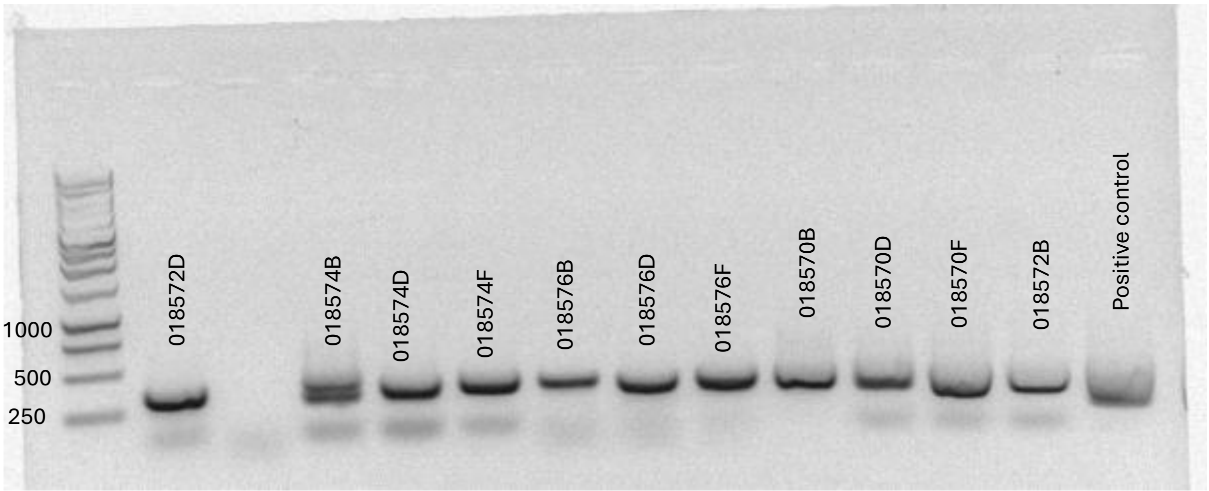
1000
500
250
018572D
018574B
018574D
018574F
018576B
018576D
018576F
018570B
018570D
018570F
018572B
Positive control

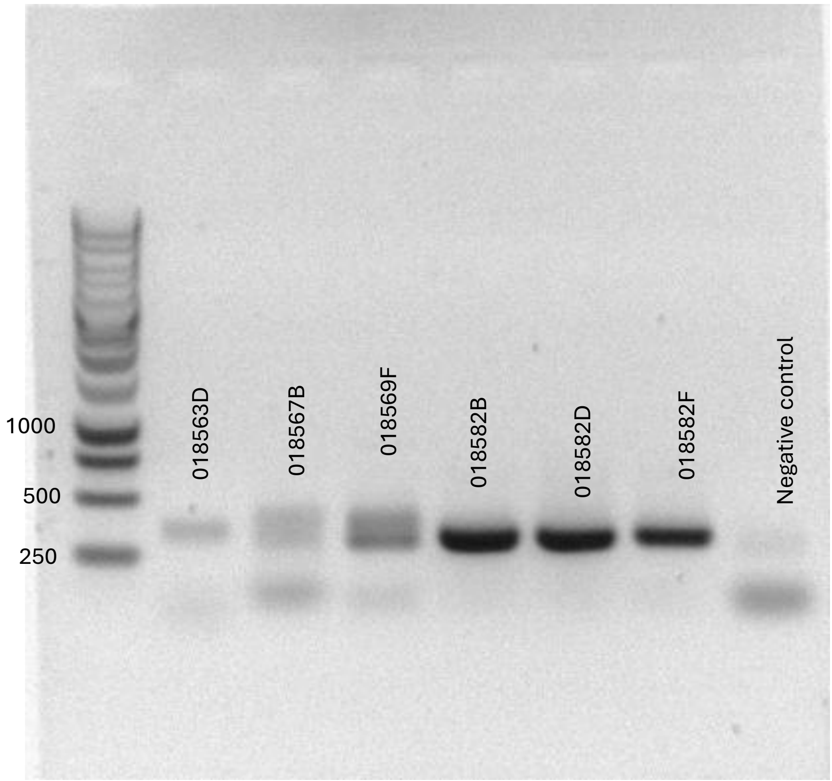
1000
500
250
018563D
018567B
018569F
018582B
018582D
018582F
Negative control

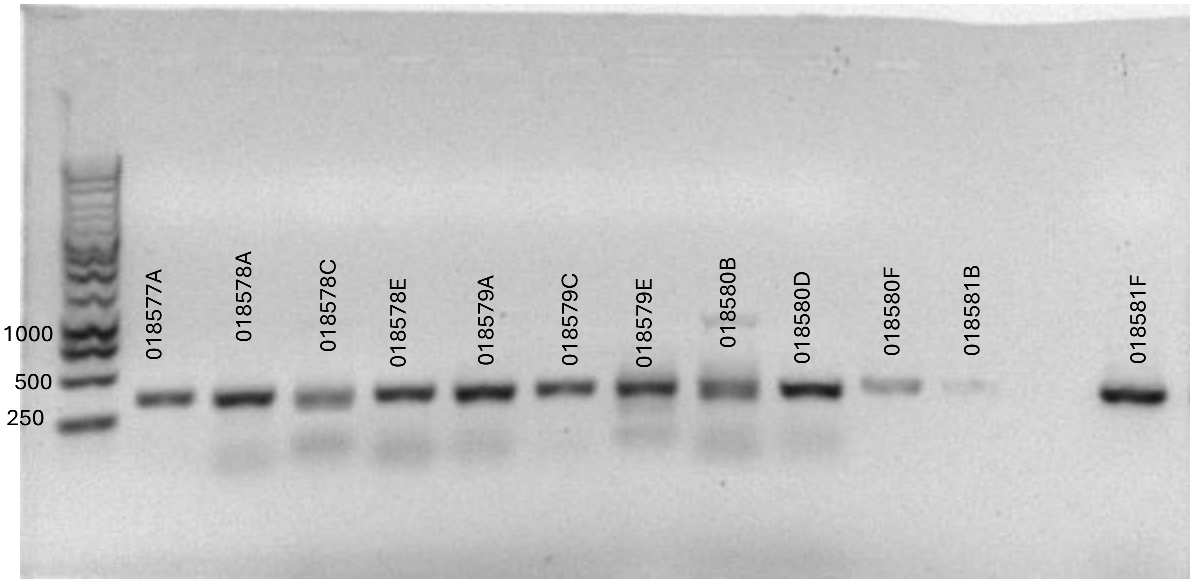
1000
500
250
018577A
018578A
018578C
018578E
018579A
018579C
018579E
018580B
018580D
018580F
018581B
018581F

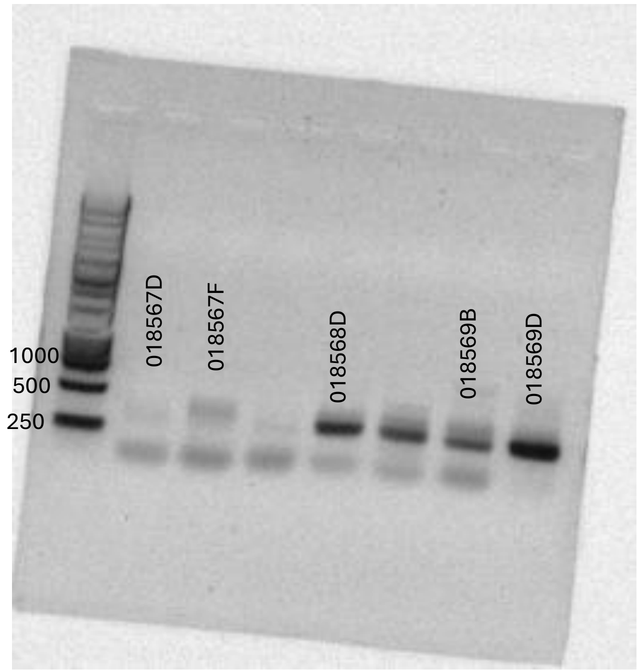
1000
500
250
018567D
018567F
018568D
018569B
018569D

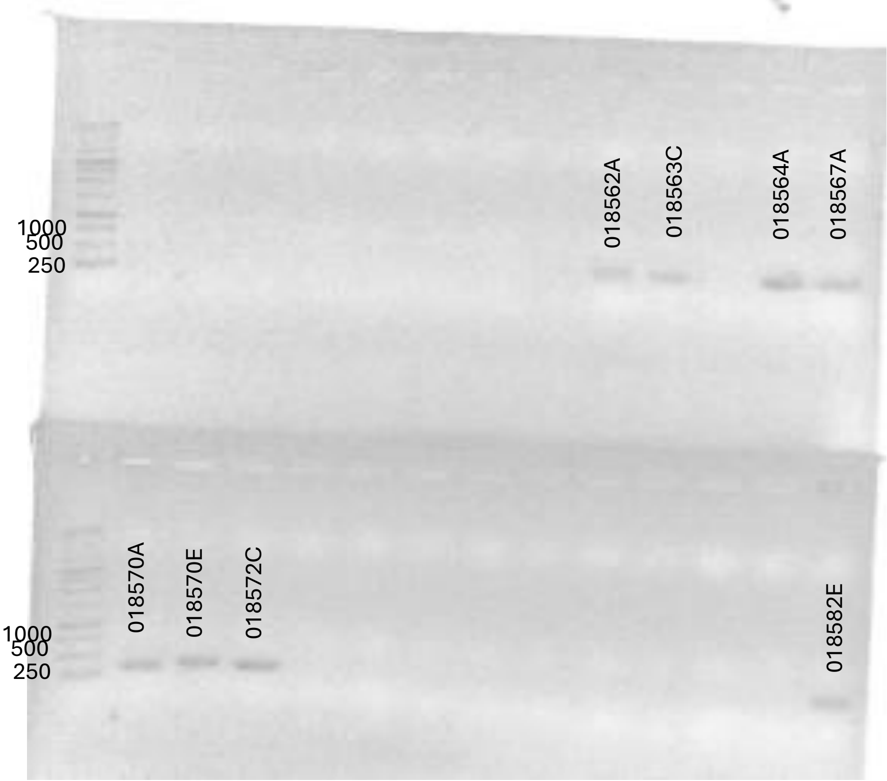
1000
500
250
018562A
018563C
018564A
018567A
1000
500
250
018570A
018570E
018572C
018582E

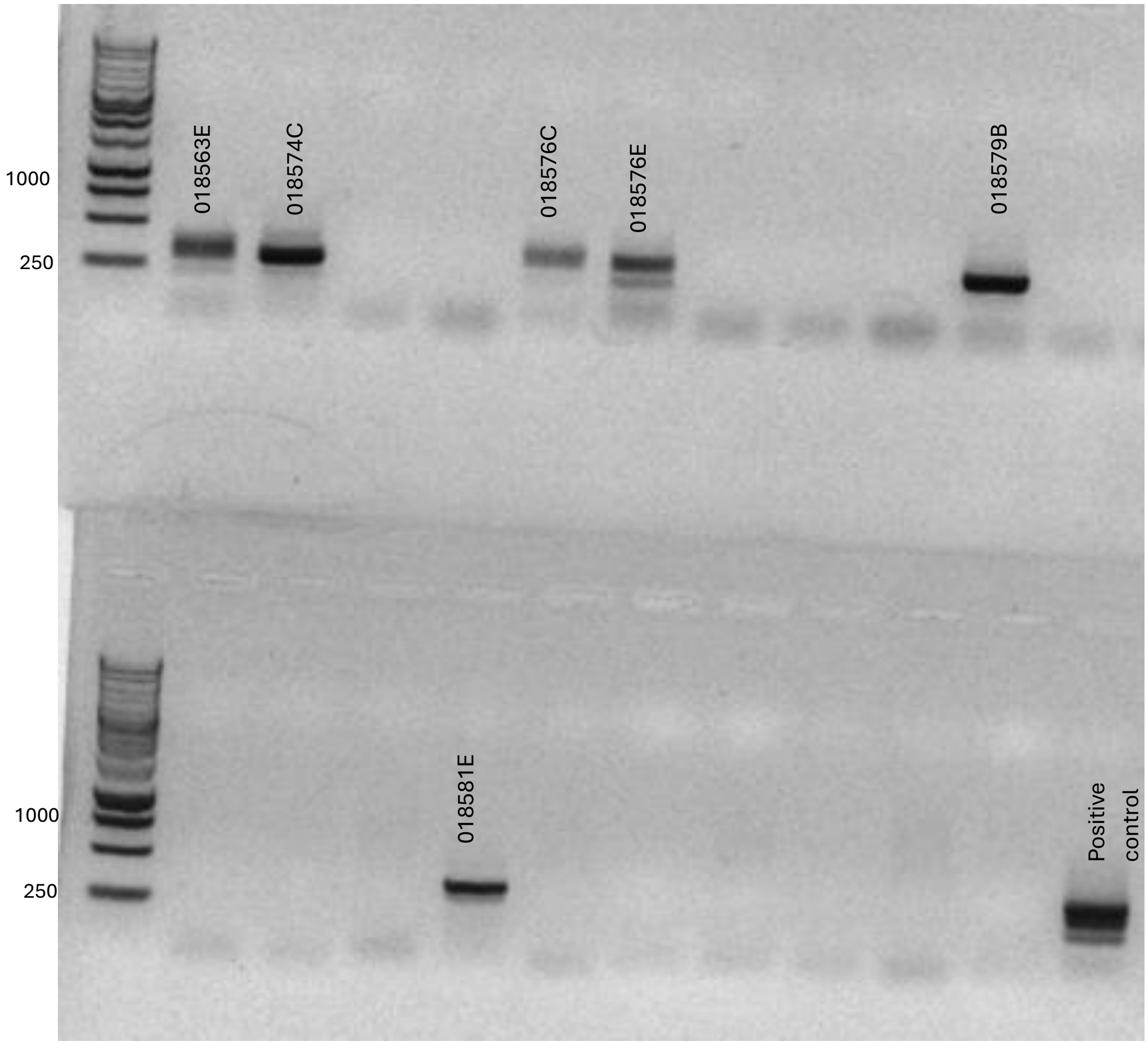
1000
250
018563E
018574C
018576C
018576E
018579B
1000
250
018581E
Positive control

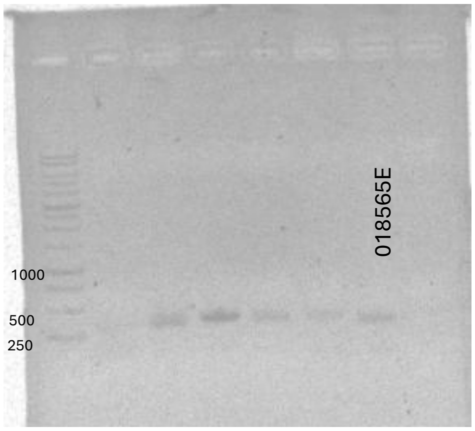
1000
500
250
018565E

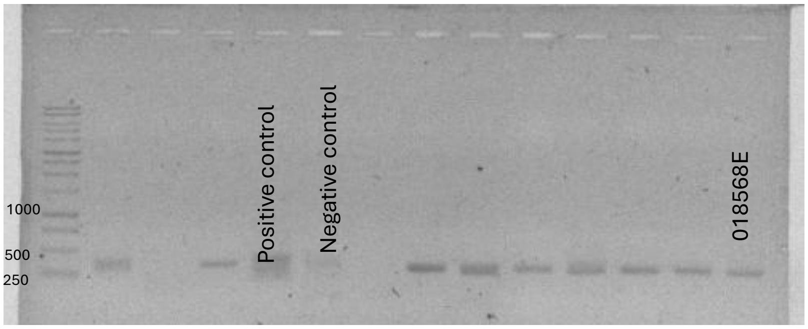
1000
500
250
Positive control
Negative control
018568E

Transect B

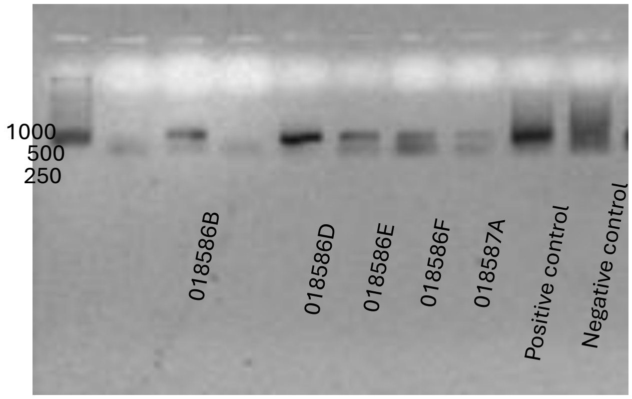
1000
500
250
018586B
018586D
018586E
018586F
018587A
Positive control
Negative control

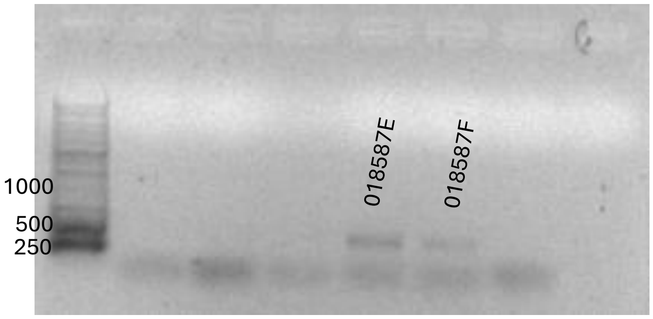
1000
500
250
018587E
018587F

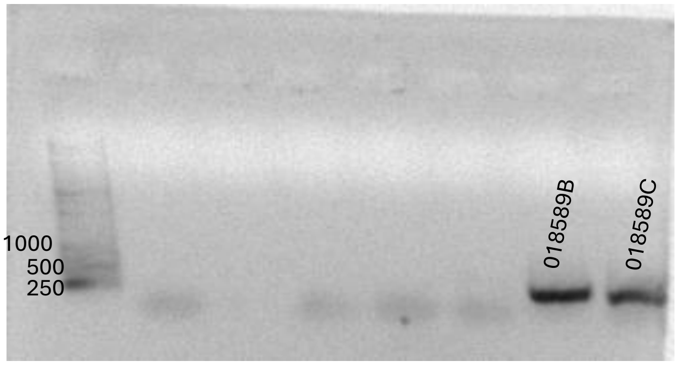
1000
500
250
018589B
018589C

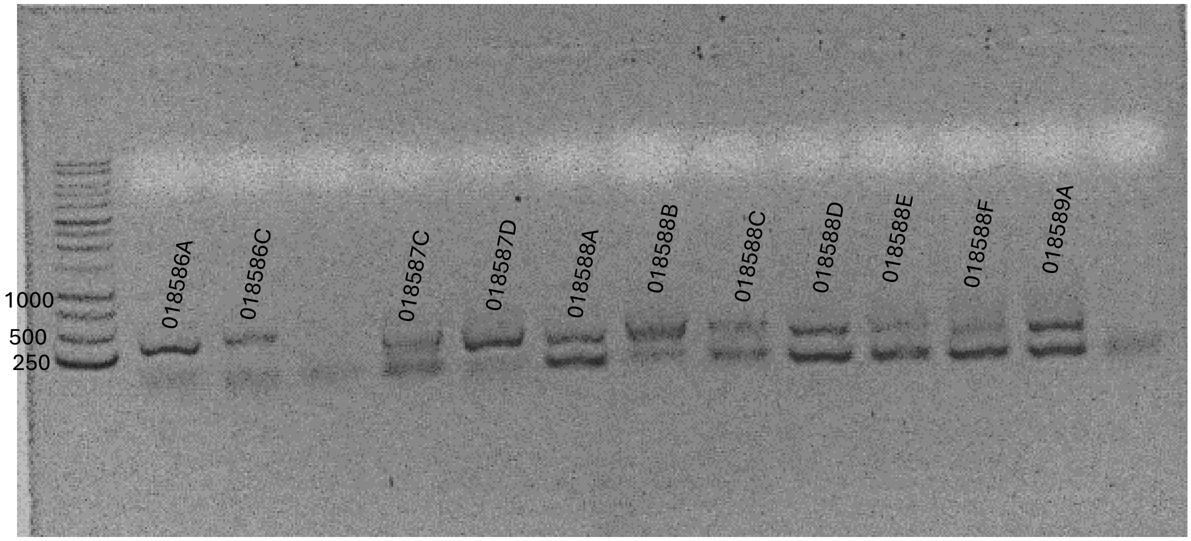
1000
500
250
018586A
018586C
018587C
018587D
018588A
018588B
018588C
018588D
018588E
018588F
018589A

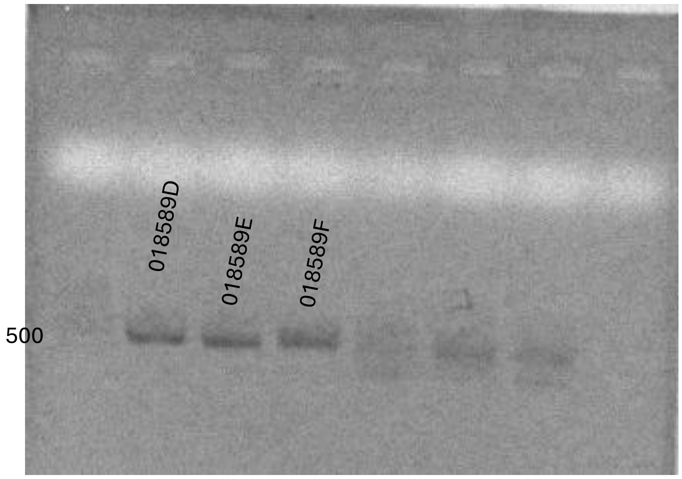
018589D
018589E
018589F
500

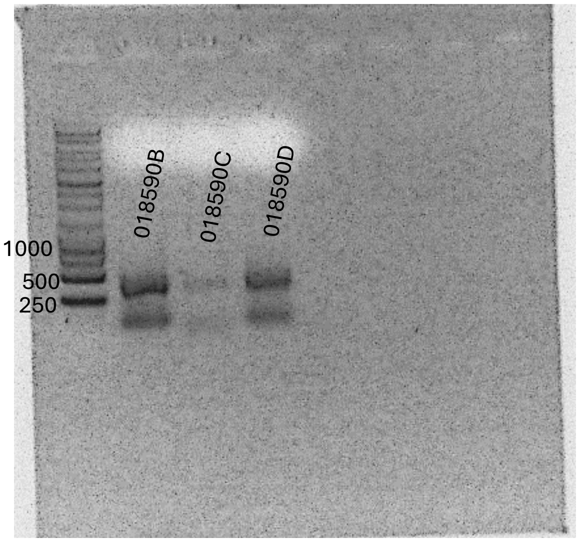
018590B
018590C
018590D
1000
500
250

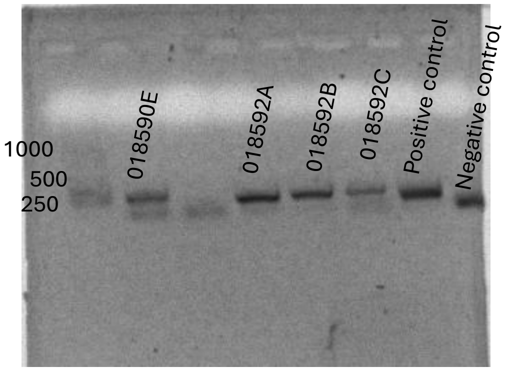
018590E
018592A
018592B
018592C
Positive control
Negative control
1000
500
250

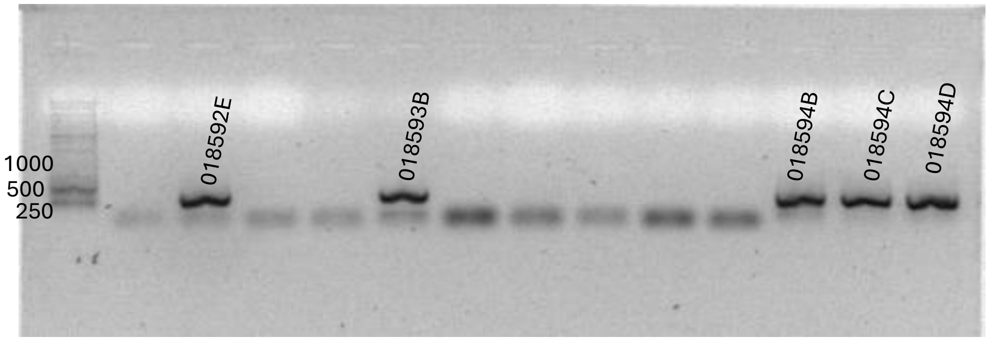
1000
500
250
018592E
018593B
018594B
018594C
018594D

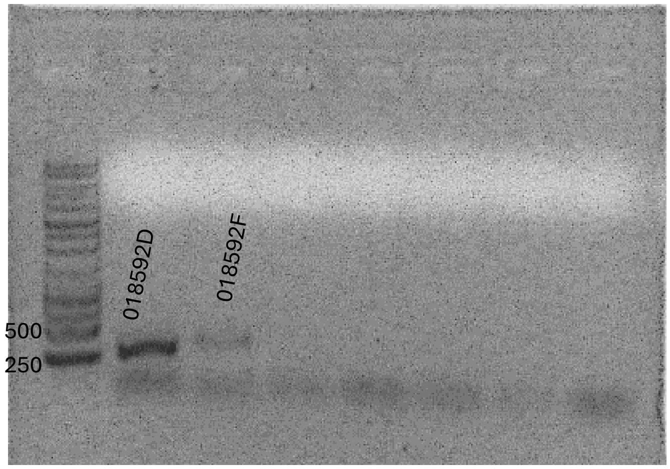
500
250
018592D
018592F

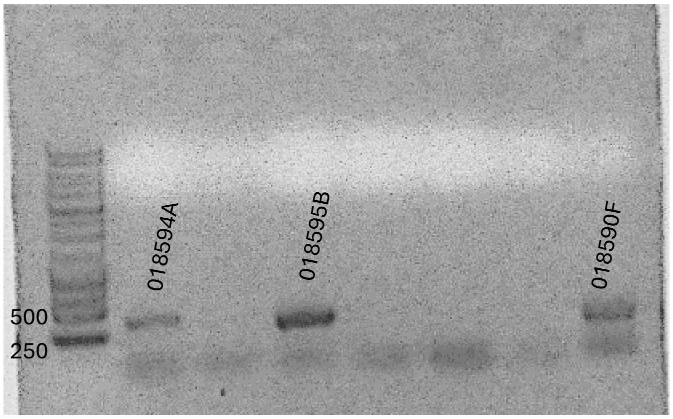
500
250
018594A
018595B
018590F

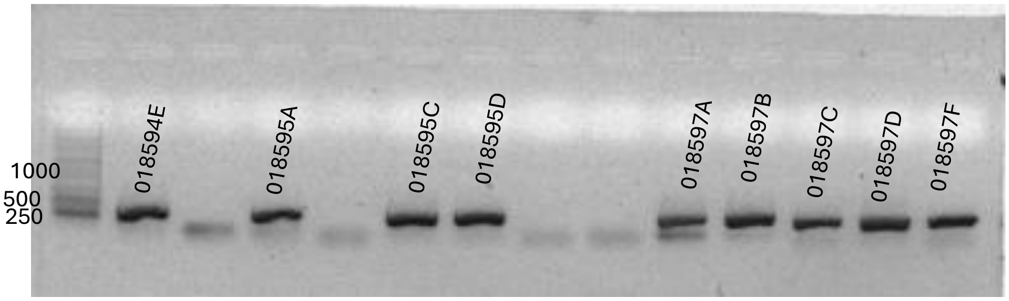
1000
500
250
018594E
018595A
018595C
018595D
018597A
018597B
018597C
018597D
018597F

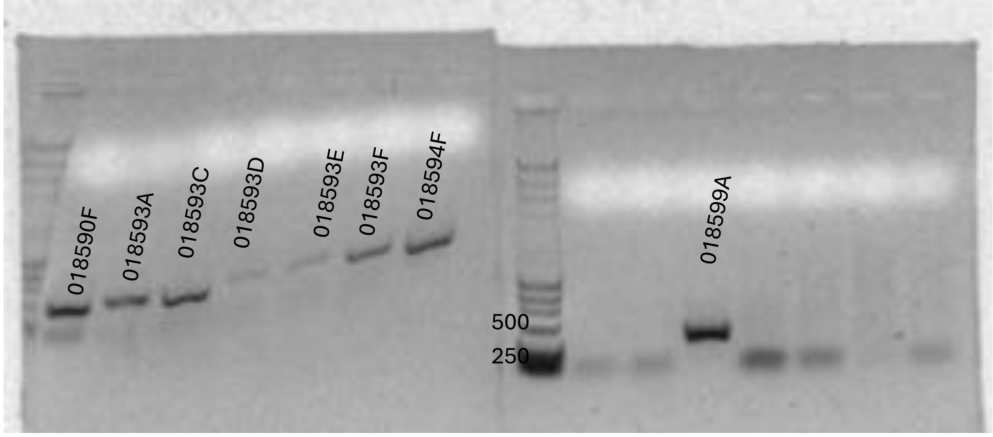
018590F
018593A
018593C
018593D
018593E
018593F
018594F
018599A
500
250

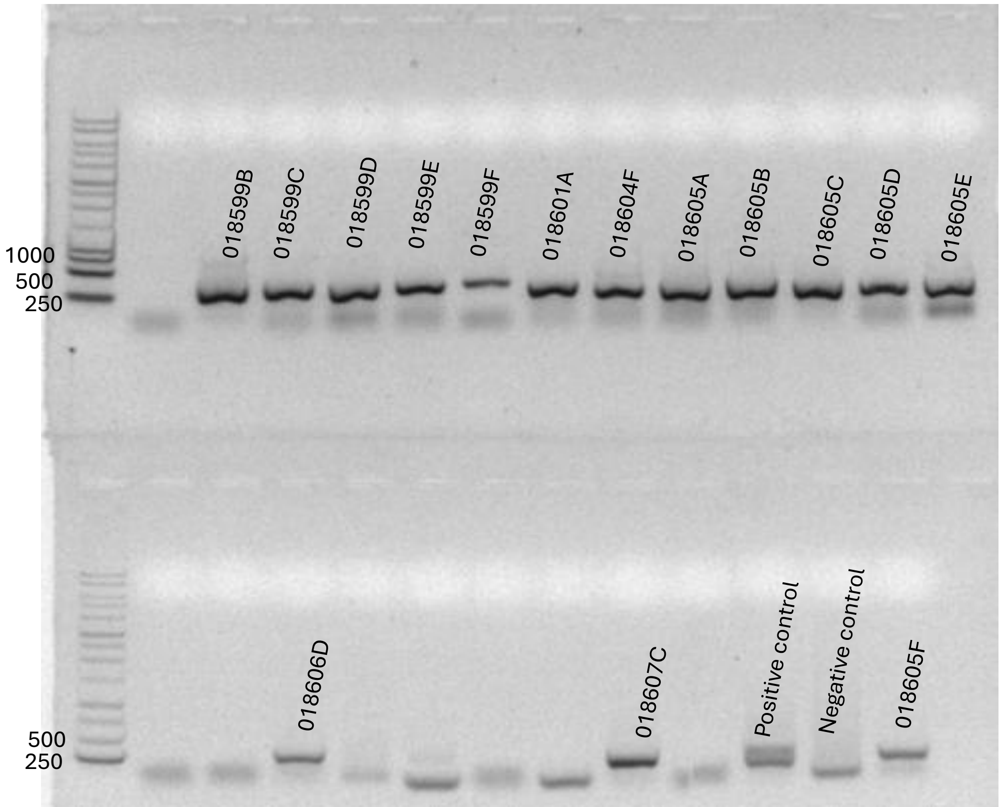
1000
500
250
018599B
018599C
018599D
018599E
018599F
018601A
018604F
018605A
018605B
018605C
018605D
018605E
500
250
018606D
018607C
Positive control
Negative control
018605F

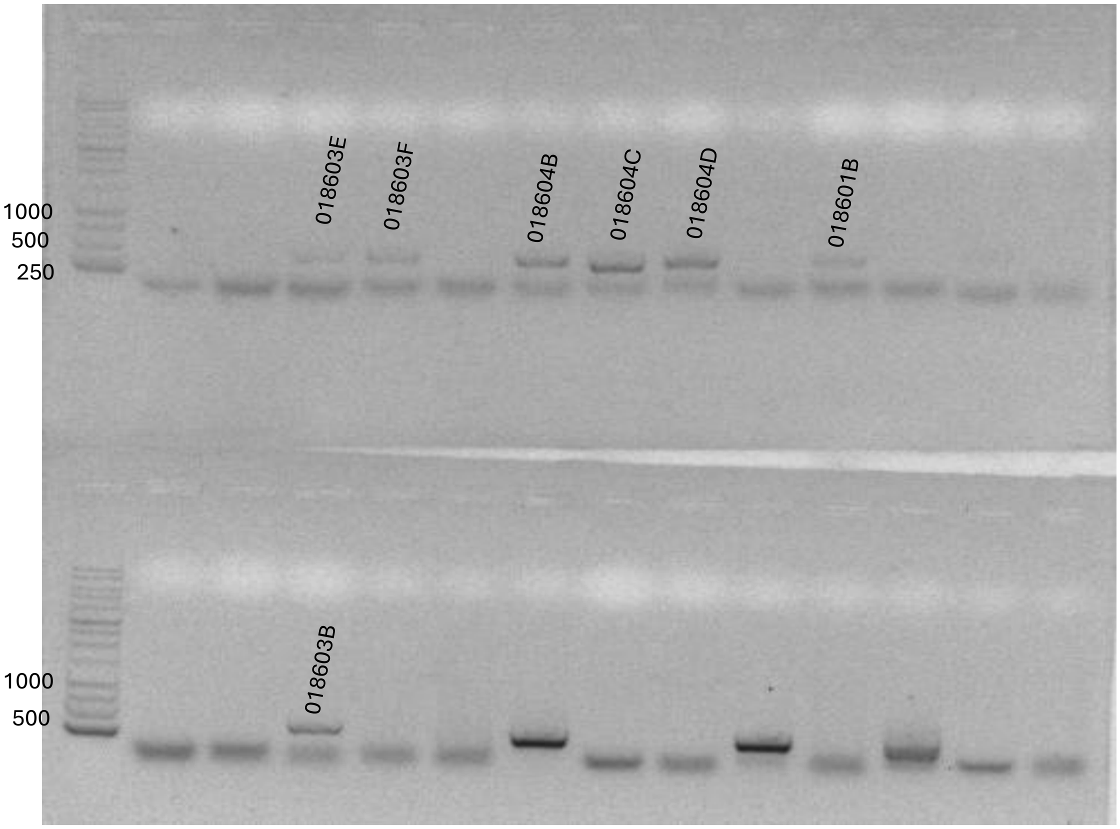
1000
500
250
018603E
018603F
018604B
018604C
018604D
018601B
1000
500
018603B

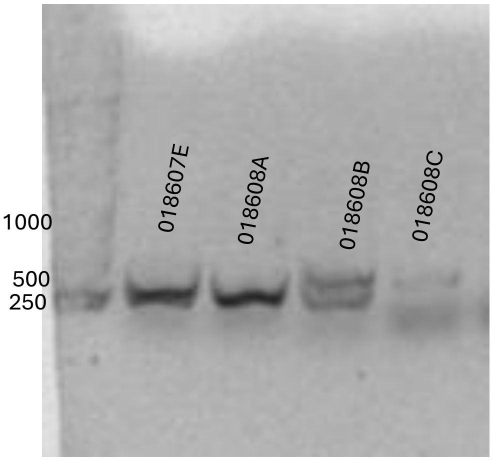
1000
500
250
018607E
018608A
018608B
018608C

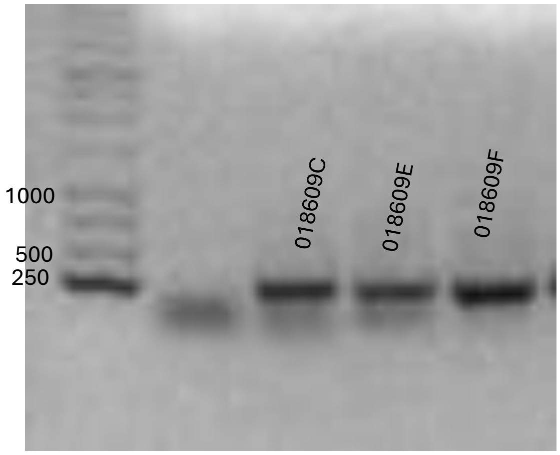
1000
500
250
018609C
018609E
018609F

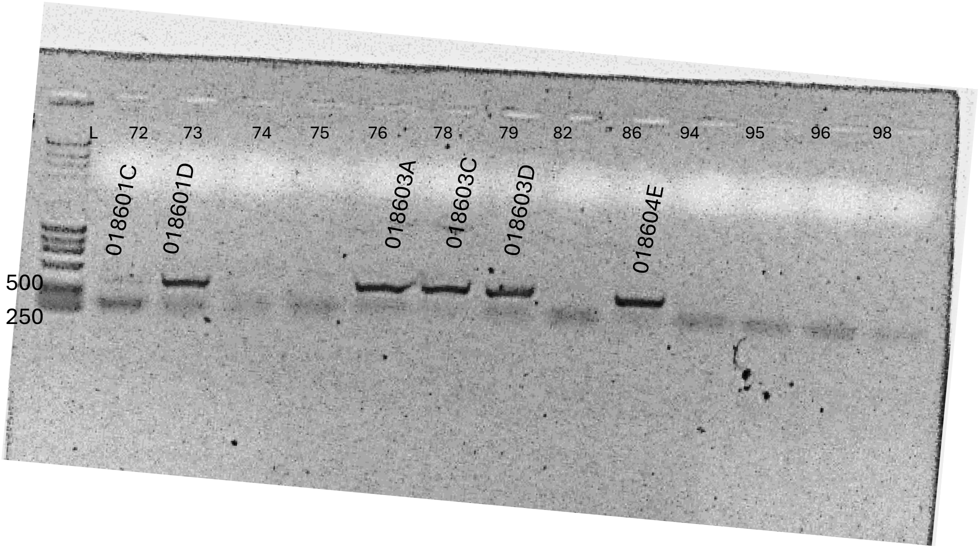
L
72
73
74
75
76
78
79
82
86
94
95
96
98
018601C
018601D
018603A
018603C
018603D
018604E
500
250

Transect C

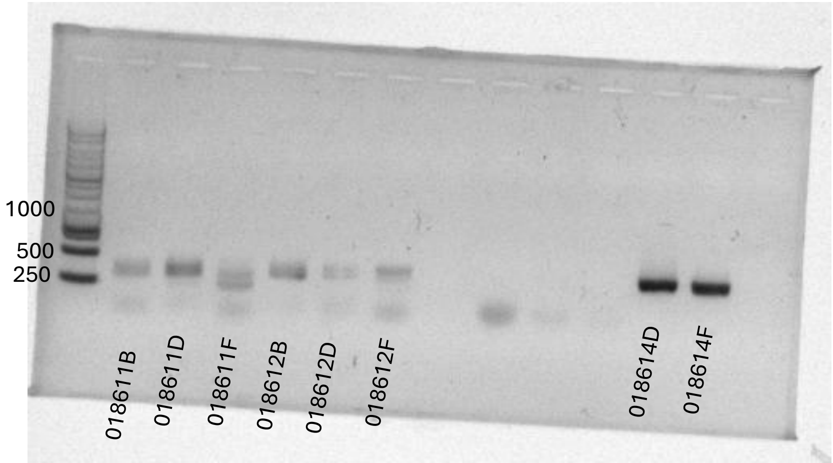

1000
500
250
018611B
018611D
018611F
018612B
018612D
018612F
018614D
018614F

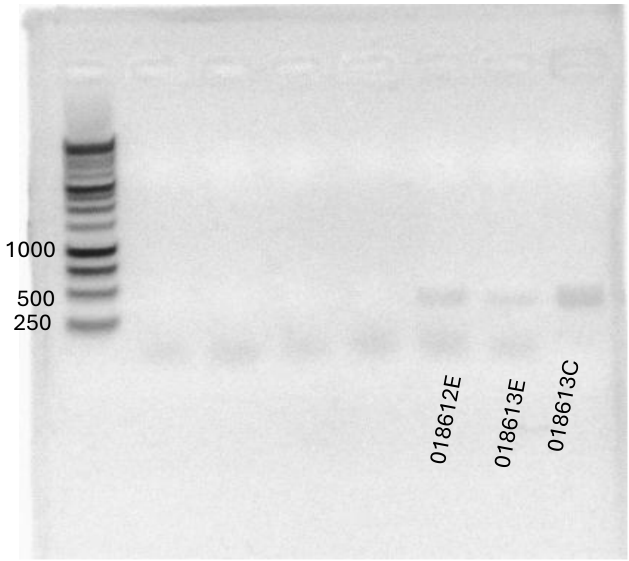

1000
500
250
018612E
018613E
018613C

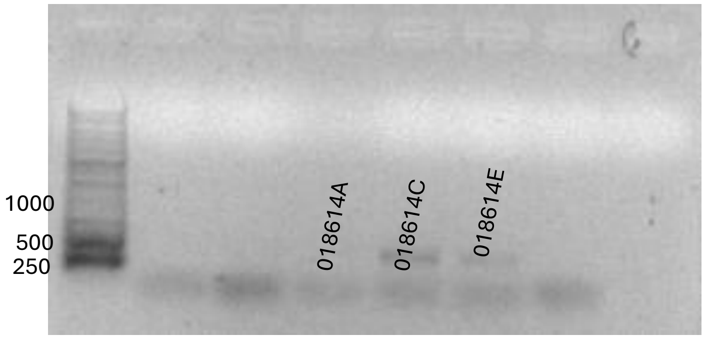
1000
500
250
018614A
018614C
018614E

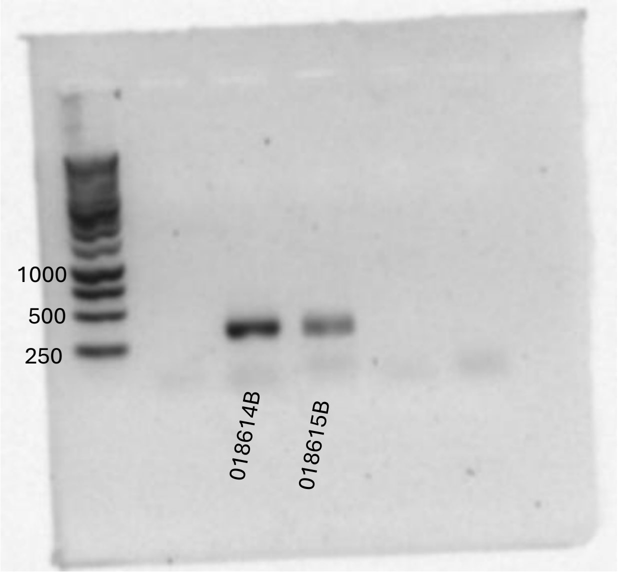
1000
500
250
018614B
018615B

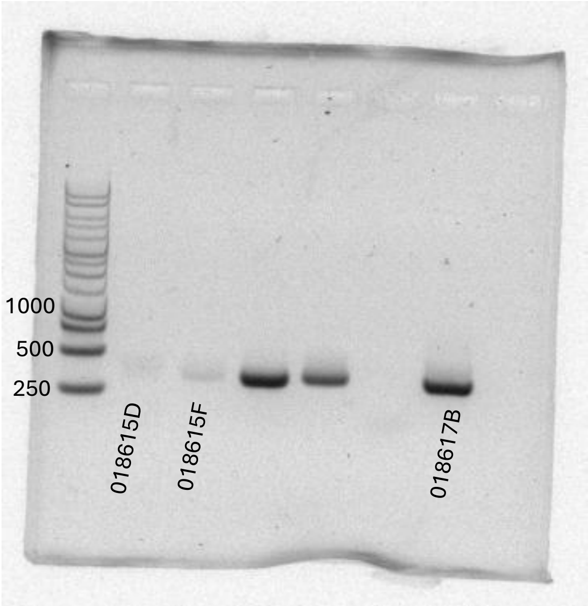
1000
500
250
018615D
018615F
018617B

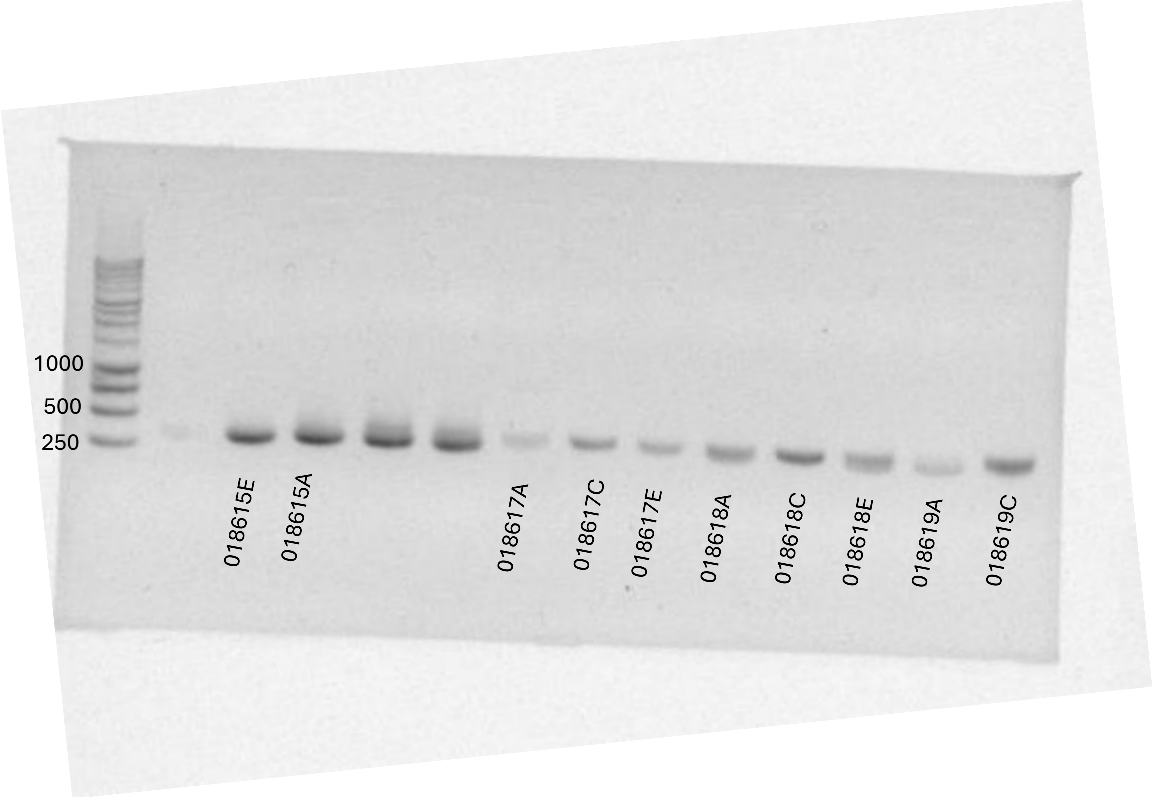
1000
500
250
018615E
018615A
018617A
018617C
018617E
018618A
018618C
018618E
018619A
018619C

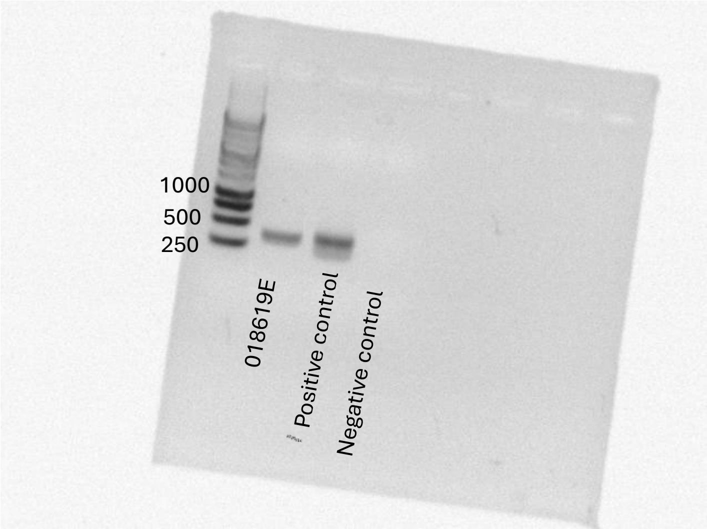
1000
500
250
018619E
Positive control
Negative control

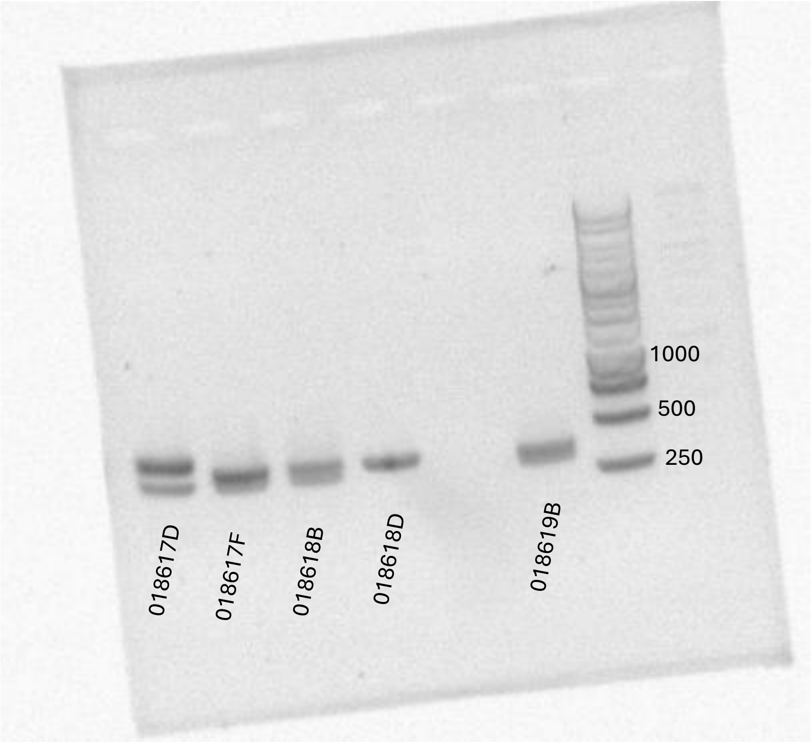
1000
500
250
018617D
018617F
018618B
018618D
018619B

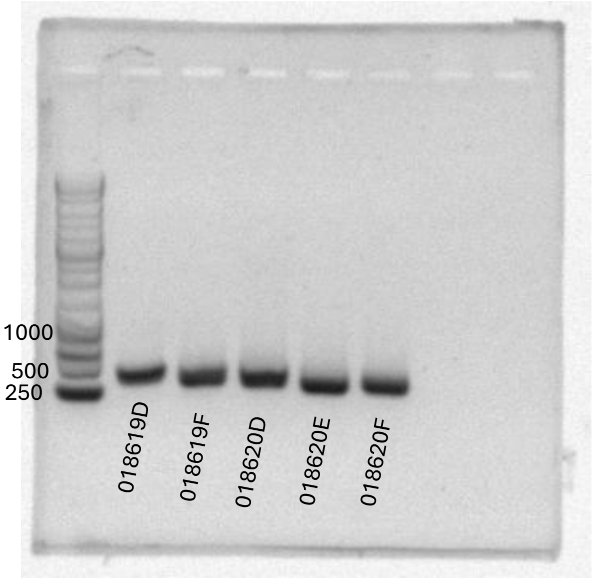
1000
500
250
018619D
018619F
018620D
018620E
018620F

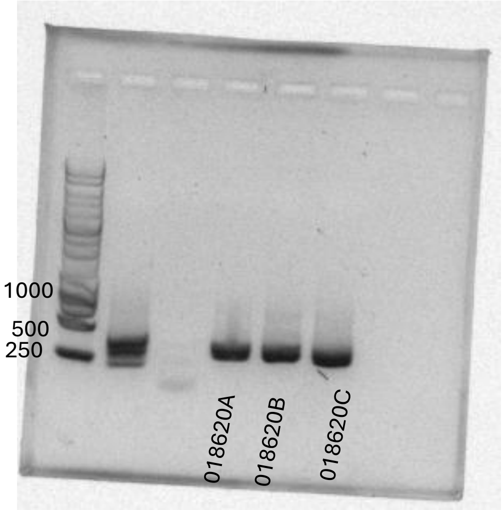
1000
500
250
018620A
018620B
018620C
